# Delayed post gadolinium MRI descriptors for Meniere’s disease: a systematic review and meta-analysis

**DOI:** 10.1007/s00330-023-09651-8

**Published:** 2023-05-12

**Authors:** Steve Connor, Mariusz T. Grzeda, Babak Jamshidi, Sebastien Ourselin, Joseph V. Hajnal, Irumee Pai

**Affiliations:** 1 School of Biomedical Engineering and Imaging Sciences, King’s College London, London, SE1 7EH UK; 2 Department of Neuroradiology, King’s College Hospital, London, SE5 9RS UK; 3 Department of Radiology, Guy’s Hospital and St Thomas’ Hospital, London, SE1 9RT UK; 4 King’s Technology Evaluation Centre, School of Biomedical Engineering and Imaging Sciences, King’s College, London, SE1 7EH UK; 5 Department of Ear, Nose and Throat Surgery, Guy’s and St Thomas’ Hospital, London, SE1 9RT UK

**Keywords:** Magnetic resonance imaging, Endolymphatic hydrops, Ear, inner, Gadolinium, Odds ratio

## Abstract

**Objectives:**

Delayed post-gadolinium magnetic resonance imaging (MRI) detects changes of endolymphatic hydrops (EH) within the inner ear in Meniere’s disease (MD). A systematic review with meta-analysis was conducted to summarise the diagnostic performance of MRI descriptors across the range of MD clinical classifications.

**Materials and methods:**

Case-controlled studies documenting the diagnostic performance of MRI descriptors in distinguishing MD ears from asymptomatic ears or ears with other audio-vestibular conditions were identified (MEDLINE, EMBASE, Web of Science, Scopus databases: updated 17/2/2022). Methodological quality was evaluated with Quality Assessment of Diagnostic Accuracy Studies version 2. Results were pooled using a bivariate random-effects model for evaluation of sensitivity, specificity and diagnostic odds ratio (DOR). Meta-regression evaluated sources of heterogeneity, and subgroup analysis for individual clinical classifications was performed.

**Results:**

The meta-analysis included 66 unique studies and 3073 ears with MD (mean age 40.2–67.2 years), evaluating 11 MRI descriptors. The combination of increased perilymphatic enhancement (PLE) and EH (3 studies, 122 MD ears) achieved the highest sensitivity (87% (95% CI: 79.92%)) whilst maintaining high specificity (91% (95% CI: 85.95%)). The diagnostic performance of “high grade cochlear EH” and “any EH” descriptors did not significantly differ between monosymptomatic cochlear MD and the latest reference standard for definite MD (*p* = 0.3; *p* = 0.09). Potential sources of bias were case-controlled design, unblinded observers and variable reference standard, whilst differing MRI techniques introduced heterogeneity.

**Conclusions:**

The combination of increased PLE and EH optimised sensitivity and specificity for MD, whilst some MRI descriptors also performed well in diagnosing monosymptomatic cochlear MD.

**Key Points:**

• *A meta-analysis of delayed post-gadolinium magnetic resonance imaging (MRI) for the diagnosis of Meniere’s disease is reported for the first time and comprised 66 studies (3073 ears).*

• *Increased enhancement of the perilymphatic space of the inner ear is shown to be a key MRI feature for the diagnosis of Meniere’s disease.*

• *MRI diagnosis of Meniere’s disease can be usefully applied across a range of clinical classifications including patients with cochlear symptoms alone.*

**Supplementary Information:**

The online version contains supplementary material available at 10.1007/s00330-023-09651-8.

## Introduction

Meniere’s disease (MD) is an inner ear disorder characterised by the clinical presentation of episodic vertigo, low- to mid-frequency hearing loss and fluctuating aural symptoms, with a potentially devastating impact on quality of life. Prevalence as high as 513/100,000 has been reported in population-based studies [1]. A series of diagnostic criteria have been proposed by international societies, most recently in 1995 and 2015 [4, 6], which are largely based on the subjective reporting of symptoms and audiometry [2–8]. However, MD may have variable manifestations, with the cardinal symptoms present in only 40% of patients with early disease [9, 10]. The ability of clinical criteria to capture atypical phenotypes [11] and to distinguish MD from alternative diagnoses has also been questioned [8, 12, 13]. Nevertheless, there have been no other reliable diagnostic methods until recently [14, 15], with the conventional role of MRI being to exclude other pathologies.

Endolymphatic hydrops (EHs) refer to the expansion of the endolymphatic space (ES) of the inner ear at the expense of the surrounding perilymphatic space (PS) and is considered to be the histological hallmark of MD [4, 16]. The MRI depiction of EH with delayed post-gadolinium MRI was first described in 2007 [17]. The ability of MRI to demonstrate EH and diagnose MD with both intra-tympanic and intravenous contrast administration has been evaluated in subsequent studies [18–87]. The utilisation of delayed post-gadolinium MRI has been a major advance in otological imaging, with increasing worldwide application and a consequent shift in the diagnostic paradigm of MD. Some recent MD classifications [7] have even incorporated MRI within the diagnostic criteria [8, 88].

The reporting of EH on MRI is generally based on the evaluation of descriptors and semi-quantitative grading scales [19, 22, 24, 31, 46, 79, 94–97] but there is little consensus on which of these perform best in distinguishing affected ears. Despite previous systematic reviews on the subject [89–93], there have been no attempts to determine the pooled diagnostic performance of MRI descriptors. Meta-analysis would provide greater certainty as to how MRI should be interpreted to optimally corroborate the diagnosis of MD. Furthermore, whilst previous systematic reviews have been applied to a narrowly defined reference clinical standard such as “definite MD”, another question relates to whether MRI is diagnostically useful in atypical or monosymptomatic forms, such as cochlear MD (cMD) and vestibular MD (vMD) [98].

This systematic review and meta-analysis aimed to determine the diagnostic performance of MR descriptors in distinguishing ears with clinical MD, and how this differs between the MD clinical subcategories.

## Method

This study applied the Preferred Reporting Items for Systematic Reviews and Meta-analyses (PRISMA) guidelines [99] and enrolled on the Prospective Register of Systematic Reviews (PROSPERO), CRD42022299285.

### Search strategy

The search strategy was based on PICOS (population; intervention; comparator; outcome; study design). Population was defined as ears with MD symptoms; intervention as delayed (3–6 h for intravenous; 24 h for intratympanic) post-gadolinium MRI; comparator (reference standard) as clinical criteria for MD; outcome as qualitative or semi-quantitative MRI descriptors for MD; and study design as case-controlled cross-sectional studies [100]. Search terms were adapted after a pilot search to include relevant synonyms, before being subjected to Peer Review of Electronic Search Strategies (PRESS) [101]. Searches were performed in MEDLINE, EMBASE, Web of Science, Scopus, Cochrane Register of Controlled Trials and LILACS databases (supplementary 1). The search was performed from 2000 onwards. The searches were finally updated on 17/02/2022. Manual forward and backward searches were performed for all eligible and review articles. The five most frequently cited journals were hand-searched (2010–2021) and grey literature interrogated. Mendeley Reference Manager was used to collate the literature and duplicate studies were manually removed.

### Selection of studies

Two independent reviewers (S.C./I.P.) applied a piloted screening tool to the titles and abstracts with the following inclusion criteria: defined MD ear disease group (supplementary 2); potential inclusion of control ears without MD; analysis of delayed post-gadolinium MRI. Case studies, review articles, foreign language literature and clearly duplicate studies were excluded. The full text was then independently assessed for eligibility by both reviewers according to the PICOS criteria. Inclusion required the extraction of 2-by-2 contingency tables, comparing the presence of MRI descriptors in MD ears (supplementary 3) with either asymptomatic ears contralateral to MD, asymptomatic ears in other subjects, or ears with an alternative audio-vestibular condition. Reasons for exclusions are listed in supplementary 4. Discrepancies were resolved by discussion. Authors were contacted to address any missing data from conference abstracts and full papers, and for clarification regarding potential overlapping data.

### Data extraction

The same reviewers independently extracted data regarding (a) study characteristics: authors, year of publication, study centre and period, retrospective v prospective, sample size of MD and controls, gadolinium concentration, agent and route of administration, MRI system strength and sequences; (b) control type; (c) demographic and clinical characteristics: age and sex of the MD group, duration of MD, unilateral or bilateral MD, and clinical diagnostic criteria; and (d) MRI descriptors or grading scale analysed and number of observers.

Contingency tables (2-by-2) were constructed comparing the presence of clinical MD (reference test) to the presence of each MRI descriptor (index test).

### Quality assessment

The methodological quality of the eligible studies was evaluated with a tailored Quality Assessment of Diagnostic Accuracy Studies version 2 (QUADAS-2) tool [102] by the two reviewers independently. Review specific guidance was developed with respect to the signalling questions (supplementary 5).

### Statistical analysis

Bivariate diagnostic random-effects meta-analysis was conducted with R 4.2.1 (package “meta”) to evaluate the diagnostic performance of each MRI descriptor. The results were tabulated with receiver operating curve (ROC) plots and corresponding forest plots [103]. Sensitivity, specificity, diagnostic odds ratio (DOR), area under the curve, and positive and negative likelihood ratios were calculated after pooling of true positive, true negative, false positive and false negative values. Heterogeneity was assessed using Cochran’s *Q* test which tests the equality of sensitivities and specificities among the studies based on a chi-square distributed statistic (*p* < 0.001).

Meta-regression used a random effects model with restricted maximum likelihood estimation and the Kruskal–Wallis test evaluated (*p* < 0.05) differences in sensitivity and specificity between subgroups. The diagnostic performance of MRI descriptors for distinguishing ears with MD according to the current reference standard of the 2015 Barany criteria (“definite 2015”) was compared with that for each of the other clinical classifications: “definite 1995”, “probable 2015”, “probable 1995”, “possible 1995”, “cMD” and “vMD” (supplementary 2). Subgroup analysis of diagnostic performance for each MRI descriptor was performed for the “definite 2015” category, those clinical classifications in which diagnostic performance significantly differed (*p* < 0.05) and any other mono-symptomatic clinical classifications.

The following variables were also analysed for their potential influence on diagnostic performance: (a) control group type (asymptomatic ears contralateral to MD v asymptomatic ears in other subjects v ears with other audio-vestibular conditions); (b) route of gadolinium administration (IV v IT); (c) number of image reviewers (single v multiple observers); (d) analysed on an ear basis v patient basis; (e) sequences or post-processing depicting different bone signal (intermediate v low); (f) low risk of bias (any domain vs none); (g) high applicability (any vs none); (h) study design (prospective v other, consecutive recruitment v other). Deek’s funnel plots [104] depicted publication bias and sample size effect.

## Results

### Systematic review

**Fig. 1:**
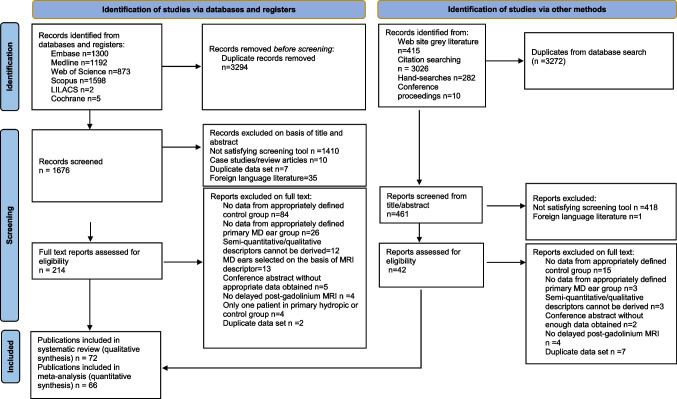
Flow chart summary of the literature search and systematic review process

Figure 1 is a study flow diagram documenting the search results and reasons for exclusion at each stage. The screening tool indicated 256 potentially relevant articles. After full text review, 72 studies were considered eligible.

### Study characteristics

The characteristics of all eligible studies are documented in Table 1 and supplementary 6 [12–87]. There was a total of 3073 MD ears (mean age 40.2–67.2 years). The clinical classifications applied were as follows (supplementary 2): “definite MD” (2015, *n* = 37; 1995 *n* = 23), “probable MD” (2015, *n* = 13; 1995, *n* = 16), “possible MD” (*n* = 16), “cMD” (*n* = 11) and “vMD” (*n* = 7) (Table 1). The commonest control group type was asymptomatic ears contralateral to MD (*n* = 59) (Table 1). Gadolinium administration was described as intravenous (*n* = 57), intra-tympanic (*n* = 14) or both (*n* = 1), whilst MRI was most frequently performed at 3 T (*n* = 71) and with a 3D FLAIR sequence (*n* = 62) (supplementary 6). Multiple observers were documented in 40/72 studies, with inter-observer agreement statistics presented in 18/72 (kappa range 0.59–0.93) (supplementary 6). After clarification with authors, six studies were deemed to have partly overlapped data sets (supplementary 6). The meta-analysis therefore included 66 unique studies.

**Table 1:** Study characteristics

	**Study design:** Prospective (P) Retrospective (R) Uncertain (U)/ Consecutive(C) Non-consecutive (N) Uncertain (U)	**Number of ears:**MD ears/control ears	**Age of MD patients:** Average years, ± SD, [IQR], (range)	**Sex of MD ears:** Male (m)/ female (f)	**Unilateral MD ears/ bilateral MD ears (vMD patients**^**α**^**) and duration of MD in months (average and range)**	**Clinical classification defining MD ears:** See supplementary 2	**Control ears #:**Asymptomatic MD ear (asymp), normal ear (normal), other condition (cond)
**Attye (2017**^**a**^**) **[18]	R/C	200/260	67.2 ± 11.7	67 m/133 f	200/0 NS	Definite 2015	200 asymp /60 normal
**Attye (2017**^**b**^**) **[19]	U/C	30/60	54.2 ± 12.9	15 m/15 f	30/0 NS	Definite 1995	Normal
**Attye (2018) **[20]	P/C	60/80	53.7	29 m/31 f	40/0 (20) NS	20 definite 2015/ 20 probable 1995 (also vestibular MD)/ 20 cochlear MD	20 asymp/ 60 normal
**Attye (2020) **[21]	P/C	20/60	54 (52–67)	11 m/9 f	20/0 NS	Definite 2015	20 asymp/ 40 normal
**Barath (2014) **[22]	R/C	61/45	27–72	42 m/21 f	45/16 NS	43 definite 1995/ 3 probable 1995/ 15 possible 1995	Asymp
**Beckers (2016) **[23]	R/U	25/21	NS	NS	21/4 NS	Definite 2015	Asymp
**Bernaerts (2019) **[24]	R/C	78/78	NS	NS	78/0 NS	Definite 2015	Asymp
**Bernaerts (2022) **[25]	R/C	29/29	58.8	13 m/16 f	29/0 58 ± 67	Definite 2015	Asymp
**Boegle (2021) **[26]	U/C	75/66	55.2 ± 14.9	39 m/36 f	56/19 56.7	35 definite 2015/ 40 probable 2015	Normal
**Carfrae (2008) **[27]	U/U	7/7	52–72	NS	7/0 NS	Definite 1995	Asymp
**Chen (2021) **[28]	P/U	70/70	50.2	29 m/41 f	70/0 NS	Definite 2015	Asymp
**Choi (2017) **[29]	P/U	16/16	47.9 (21–65)	12 m/4 f	16/0 NS	Definite 1995	Asymp
**Connor (2020) **[30]	R/C	22/22	50.4 (31–79)	5 m/17 f	22/0 60 (6–300)	16 definite 2015/ 6 probable 2015	Asymp
**Conte (2018) **[31]	R/C	27/71	60.6 ± 18.6	15 m/10 f	23/4 77 (12–144)	Definite 2015	23 asymp/ 24 normal/ 24 cond (SoSNHL)
**Dominguez (2021) **[32]	R/U	155/151	40.9	62 m/83 f	121/0 (34) 64 ± 72	83 definite 2015/ 38 cochlear MD/ 34 vestibular MD	121 asymp/ 15 normal/ 15 cond (SoSNHL)
**Eliezer (2017) **[33]	U/C	6/12	54.6 [42; 76]	3 m/3 m	6/0 NS	Definite 2015	Normal
**Eliezer (2018) **[34]	R/U	20/20	52.0	8 m/12 f	20/0 NS	12 definite 1995/ 2 probable 1995/ 6 possible 1995	Asymp
**Eliezer(2021) **[35]	R/U	30/30	55.6 [41.6; 69.6]	11 m/19 f	30/0 NS	Definite 1995 or probable 1995 or possible 1995^˄^	Asymp
**Eliezer(2022) **[36]	R/U	39/19	57.0 (29.0–77.0)	18 m/21 f	19/0 (20) NS	19 definite 2015/ 20 vestibular MD	Cond (VM)
**Fang (2012) **[37]	U/U	31/50	NS	NS	NS NS	Definite 1995 or probable 1995 or possible 1995^˄^	34 asymp or normal/ 16 cond (SoSNHL)
**Fiorino (2011) **[38]	U/U	26/9	56 (25–78)	18 m/8 f	26/0 36 (6–108)	Definite 1995	Asymp
**Grieve (2012) **[39]	P/U	11/2	50.1 ± 18.2 (19–76)	NS	11/0 NS	Definite 1995 or probable 1995 or possible 1995^˄^	Normal
**Grosser (2021) **[40]	R/C	105/67	NS	NS	NS NS	Definite 2015	Asymp
**Hagiwara (2014) **[41]	U/U	11/19	31–72	5 m/5 f	9/1 NS	Definite 1995	9 asymp/ 10 normal
**Horii (2011) **[42]	U/U	9/8	48.8 (32–66)	4 m/5 f	9/0 NS	Japanese criteria 2011^˄^	Cond (SoSNHL)
**Imai (2017) **[43]	R/U	35/35	53 (22–77)	13 m/22 f	35/0 NS	Definite 1995	Asymp
**Ito(2016) **[44]	U/U	50/62	59.8 (22–81)	15 m/17 f	32/18 NS	Definite 1995 or probable 1995 or possible 1995^˄^	32 asymp/ 30 normal
**Jasinska (2022) **[45]	U/U	38/38	54.39	19 m/19 f	38/0 93 (6–252)	Definite 2015	Asymp
**Kahn (2020) **[46]	R/U	35/50	54.5 ± 13.9	14 m/17 f	27/8 95 ± 83	Definite 2015	27 asymp/ 23 normal
**Katayama (2010) **[47]	U/U	32/4	55.0	14 m/14 f	24/6 (2) NS	21 definite 1995/ 8 possible 1995 including 2 vestibular MD/ 3 cochlear MD	Cond (SoSNHL)
**Kawai (2010) **[48]	R/U	16/2	51.3 (16–67)	9 m/6 f	NS NS	17 NS^˄^ 1 cochlear MD	Cond (SoSNHL)
**Kenis (2021) **[49]	U/U	9/9	62.9 (49–77)	6 m/3 f	9/0 NS	5 definite 2015/ 4 probable 2015	Asymp
**Kierig (2019) **[50]	U/U	23/47	53.1 ± 16.7 (22–77)	14 m/9 f	NS NS	NS^˄^	24 normal/ 23 cond (VM)
**Li (2020) **[51]	U/U	178/178	52 (23–74)	110 m/68 f	178/0 44 (2–240)	Definite 1995	Asymp
**Li (2022) **[52]	P/U	19/19	47.21 ± 5.39	9 m/10 f	19/0 NS	Definite 2015	Asymp
**Lin (2021) **[53]	U/N	12/12	52 ± 15	12 m/10f	12/0	Definite 2015 or probable 2015^˄^	Asymp
**Liu (2015) **[54]	P/U	30/30	49.2 (24–66)	20 m/10 f	30/0 2–480	30 definite 1995 or probable 1995 or possible 1995^˄^	Asymp
**Mainnemarre (2020) **[55]	R/U	56/38	51.8 ± 13.3	16 m/9 f	38/18 NS	Definite 1995 or probable 1995 or possible 1995^˄^	Asymp
**Morimoto (2017) **[56]	U/U	53/29	51.4	16 m/13 f	29/24 125.6	Definite 1995	Asymp
**Morimoto (2020) **[57]	U/U	26/69	NS	NS	22/4 NS	Definite 2015	42 asymp/ 27 cond (SoSNHL)
**Morita (2020) **[58]	U/U	46/30	46.6 (19–83)	17 m/229 f	30/0 (16) 33 (2–408)	30 definite 2015 16 vestibular MD	Asymp
**Murofushi (2020) **[59]	U/U	14/14	53.8 (24–76)	4 m/10 f	14/0 NS	8 definite 2015/ 6 probable 2015	Asymp
**Naganawa (2012**^**a**^**) **[60]	U/C	12/8	49 (27–67)	6 m/4 f	8/4 NS	9 2008 Japanese criteria^˄^/ 3 cochlear MD	Asymp
**Naganawa (2012**^**b**^**) **[61]	U/U	18/8	60 (17–75)	3 m/11 f	10/6 (2) NS	11 NS/ 5 cochlear MD / 2 vestibular MD	Asymp
**Naganawa (2013) **[62]	U/C	7/4	47.2 (32–72)	4f/1 m	3/4 NS	1 definite 1995 6 probable 1995	3 asymp/ 1 normal
**Nahmani (2020) **[63]	R/U	19/13	46 [43; 52] (35–68)	6 m/10 f	13/6 NS	Definite 1995 or probable 1995 or possible 1995^˄^	Asymp
**Nakada (2014) **[12]	U/N	7/7	47 ± 16	2 m/5 f	0/0/ (7) NS	7 vestibular MD 1972	Cond (VM)
**Noh (2021) **[64]	U/U	16/16	47.3 ± 8.1	7 m/9 f	16/0 53 ± 63.6	11 definite 2015/ 5 probable 2015	Asymp
**Oh (2021) **[65]	P/U	29/25	61.1 ± 17.1	10 m/19 f	NS 73 ± 42	15 definite 2015/ 14 probable 2015	Cond (VM)
**Okazaki (2017) **[66]	R/C	56/114	50.4	28 m/28 f	56 113	28 definite 2015/ 9 probable 2015/ 19 probable 1995 or possible 1995	56 asymp/ 29 normal/ 29 cond (SoNHL)
**Pai (2020) **[67]	R/C	30/21	51 (30–78)	10 m/18 f	26/4 60 (6–300)	24 definite 2015/ 6 probable 2015	Asymp
**Pakdaman (2016) **[68]	R/U	32/54	NS	15 m/17 f	32/0 NS	Definite 1995	32 asymp/ 11 normal/ 11 cond (SoSNHL)
**Perez-Fernandez (2019) **[69]	R/C	22/22	55 ± 6	13 m/9 f	22/0 84 ± 24	Definite 2015	Asymp
**Pyykko (2013) **[70]	P/C	205/45	NS	NS	NS NS	Definite 1995 or probable 1995 or possible 1995^˄^	Asymp
**Qin (2021) **[71]	R/U	21/51	54.1 ± 8.3	12 m/9 f	21/0 NS	Low-tone SoSNHL 2015 Chinese guideline^˄^	Cond (SoSNHL)
**Quatre (2019) **[72]	P/U	50/25	50.5 ± 15	15 m/26 f	32/18 NS	35 definite 2015/ 15 possible 1995	Asymp
**Sano (2012) **[73]	U/U	9/7	40.5 (34–74)	4 m/3 f	5/4 NS	1 definite 1995/ 2 probable 1995/ 5 possible 1995 1 cochlear MD	5 asymp/ 2 normal
**Shi (2018) **[74]	U/U	169/139	50.9 ± 14.1 (7–83)	72 m/82 f	139/30 NS	Definite 2015	Asymp
**Shimono (2013) **[75]	P/U	20/20	NS	NS for MD cohort	20/0 14.4 (0.3–103)	Low-tone SoSNHL 2011 Japanese criteria^˄^	Asymp
**Shiraishi (2020) **[76]	R/U	20/20	60.2 ± 11.4 (37–76)	NS	20/0 NS	Definite 2015	Asymp
**Sousa (2021) **[77]	R/U	8/8	48 (47–52)	3 m/5 f	8/0 18 (24–288)	Definite 2015	Asymp
**Suarez Vega (2019) **[78]	R/C	15/15	NS	10 m/5 f	15/0 NS	6 definite 2015/ 9 probable 2015	Asymp
**Sun(2017) **[13]	P/U	30/30	53 (20–70)	15 m/15 f	NS NS	definite 2015	Cond (VM)
**Tagaya (2011) **[79]	U/U	14/10	52.1 (36–74)	6 m/6 f	10/4 31.8 (1–78)	Definite 1995/ 4 probable 1995/ 3 possible 1995 (and cochlear MD)	Asymp
**Tanigawa (2011) **[80]	U/U	3/7	51.3 (39–59)	0 m/3 f	3/0 50.3 (2–13)	Definite 1995 or probable 1995 or possible 1995^˄^	3 asymp/ 4 normal
**Tunon Gomez (2017) **[81]	P/C	6/6	40.2 (21–67)	1 m/5 f	6/0 111.6 (36–625)	Definite 2015 or probable 2015^˄^	Asymp
**Van Steekelenburg (2016) **[82]	R/C	163/149	NS	NS	121/42 NS	149 definite 2015/ 14 probable 2015	149 asymp or normal
**Vanspauwen (2016) **[83]	R/U	13/13	54.9	NS	13/0 NS	Definite 2015	Asymp
**Wu (2016) **[84]	P/U	54/54	52 (23–74)	45 m/9 f	54/0 48 (2–480)	Definite 1995	Asymp
**Xie (2021) **[85]	P/U	126/109	56 (20–81)	50 m/67 f	108/18 59 (1–192)	Definite 2015	Asymp
**Yoshida(2018) **[86]	R/C	52/106	54.0 (27–74)	22 m/20 f	32/20 99.1 (3–480)	Definite 1995	32 asymp 74 normal
**Yoshida (2021) **[87]	U/U	16/5	53.6 (26–76)	6 m/5 f	8/8 70.1 (0–300)	4 definite 2015/ 1 probable 2015/ 11 cochlear MD	Asymp

α vMD patients are documented separately since it may not be possible to localise the laterality of MD. In these cases, a positive MRI descriptor in either ear was recorded; hence, it was analysed on a “patient basis” rather than “ear basis”

^˄^Clinical classification of MD not stated, defined as a combination of different criteria or applied less widely used clinical classification

^#^ asymp: Asymptomatic ears of Meniere’s disease or other primary hydropic ear disease patients as control; normal: normal control ears in healthy volunteers or contra-lateral asymptomatic ears of other audio-vestibular conditions as control; cond: ears in other audio vestibular condition as control

*SoSNHL* sudden onset sensorineural hearing loss; *VM* vestibular migraine; *MD* Meniere’s disease; *NS *not stated

### Categorisation of MRI descriptors

**Fig. 2:**
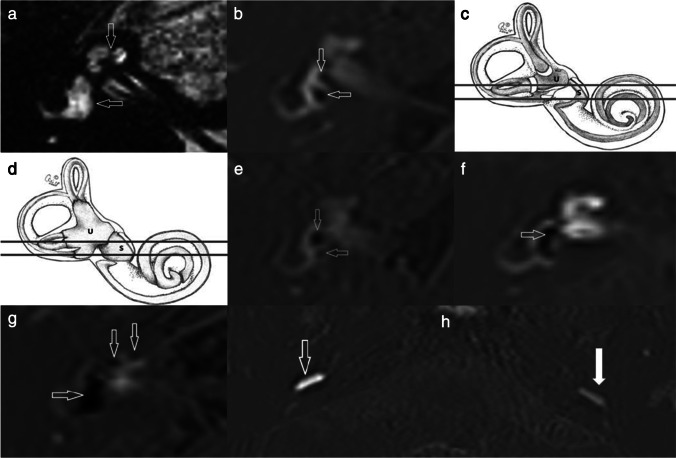
Illustrations of the MRI descriptors. **a** T2 SPACE axial image is unable to distinguish the endolymphatic from the perilymphatic space and demonstrates the inner ear structures as high signal throughout. The cochlea (vertical arrow) and the vestibule (horizontal arrow) are indicated. **b** Delayed post-gadolinium 3D REAL IR axial image in a normal ear shows that the endolymphatic structures of the saccule (vertical arrow) and the utricle (horizontal arrow) demonstrated within the vestibule with the saccule being the smaller structure. The low signal endolymph is clearly distinguished from the surrounding enhancing perilymph. Schematic representations of (**c**) the normal endolymphatic structures and (**d**) the hydropic (dilated) endolymphatic structures in a MD ear (permission to use from Miss Irumee Pai). The lines depict the level of the axial sections which encompass the utricle (U) and saccule (S) in the other images Delayed post-gadolinium 3D REAL IR axial images in **e** to **h** depict the MR descriptors in ears with MD. **e** “Saccule to utricle ratio (SURI)”. There is inversion of the saccule to utricle ratio (SURI) with the saccule (vertical arrow) being larger than the utricle (horizontal arrow). **f** “Fused utricle and saccule”. The low-signal saccule and utricle are seen to be merged (horizontal arrow). There is also borderline “ > 33% area of ES relative to total vestibular fluid area” but it does not reach “ > 50% of ES relative to total vestibular fluid area”. **g** “Enhancing PS of the vestibule not visible “and “highest grade cochlear EH”. Severe EH is demonstrated with replacement of the vestibular perilymph by non enhancing endolymph (horizontal arrow) and there is also “ > 50% of ES relative to total vestibular fluid area”. There is severe cochlear hydrops (vertical arrows) as indicated by the non enhancing cochlear duct replacing the scala vestibuli enhancement (vertical arrows). **h** A right MD ear demonstrating “increased ipsilateral perilymphatic enhancement (PLE)”. The degree of perilymphatic enhancement within the inferior segment of the right basal turn (open arrow) is increased in the right symptomatic MD ear relative to the contralateral left asymptomatic ear (filled arrow)

The two reviewers selected eleven MRI descriptors for analysis since they could be derived from at least four eligible studies. There were nine individual descriptors (Fig. 2) with two further combinations of descriptors. The presence of MRI descriptors was usually extracted from grading systems applied to the eligible studies [19, 22, 24, 46, 79, 91, 95] (supplementary 3). Due to subtle differences in some grading systems, descriptor definitions were adapted to capture the breadth of data across multiple studies (supplementary 7). Vestibular MRI descriptors of EH were as follows: “any vestibular EH”, “ > 33% area of ES relative to total vestibular fluid area” (Fig. 2F), “ > 50% area of ES relative to total vestibular fluid area” (Fig. 2G), “saccule to utricle ratio (SURI) or higher vestibular grade” (Fig. 2E),” fused utricle and saccule” (Fig. 2F) and “enhancing PS of the vestibule not visible” (Fig. 2G). Cochlear MRI descriptors of EH were “any cochlear EH” and “highest grade cochlear EH” (Fig. 2G). “Increased ipsilateral perilymphatic enhancement (PLE)” was additionally evaluated (Fig. 2H). Two MRI descriptors used a combination of features; when there was either “any vestibular EH” or “any cochlear EH”, it was termed “any EH”, and with additional increased PLE it was termed “increased ipsilateral PLE or any EH”.

### Quality of studies

**Fig. 3:**
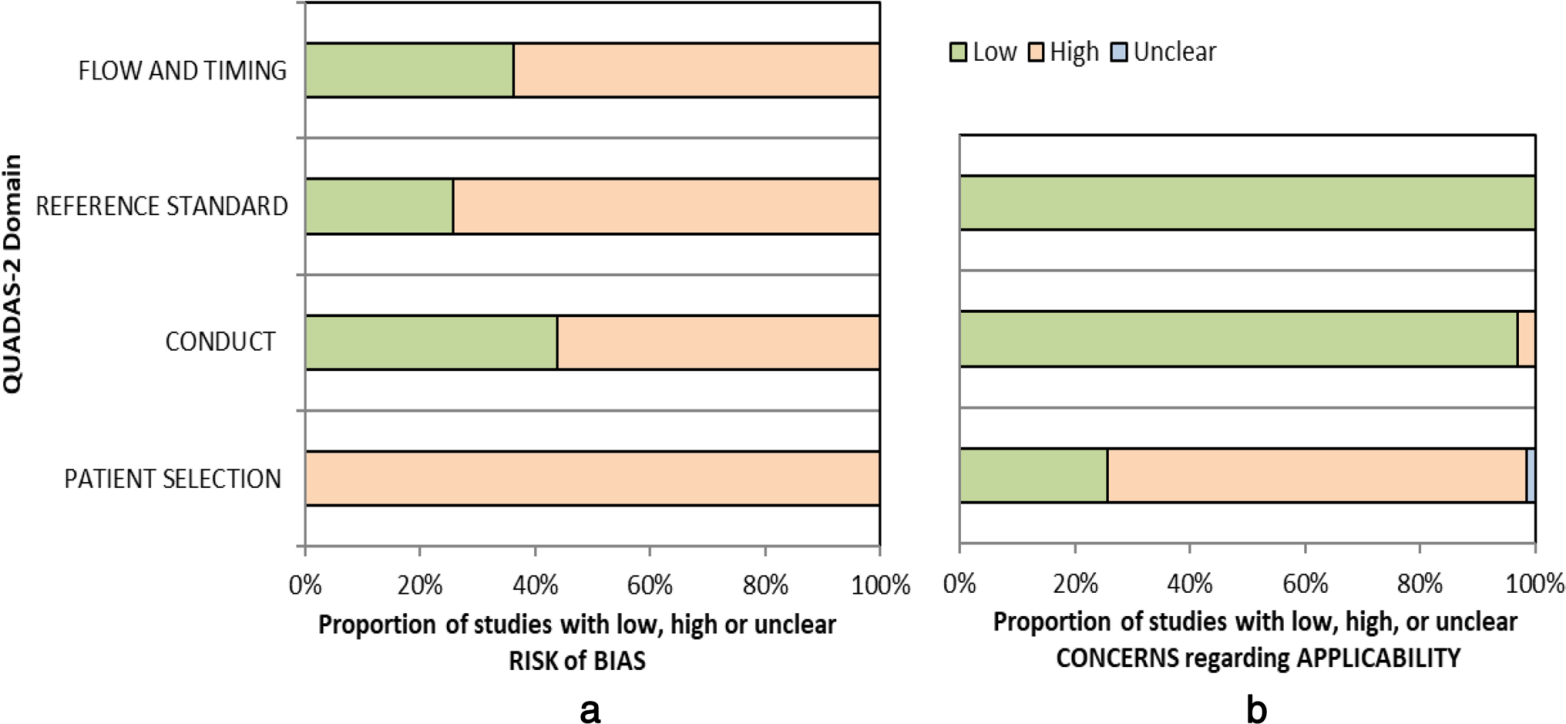
Bar charts demonstrate (**a**) the risk of bias and (**b**) applicability concerns derived from the QUADAS-2 tool for the 66 eligible studies included in the meta-analysis

QADAS-2 evaluation showed high bias across all four domains in 22/66 studies, whilst only 3/66 studies demonstrated high bias in ≤ 1 domain (Fig. 3(A)). “Patient selection” always resulted in high bias since all studies were case-controlled. High bias was reported for “conduct and interpretation of test” since most studies only analysed MD cohorts and observers could not be blinded. “Reference standard conduct and interpretation” resulted in high bias when clinical classifications other than “definite 2015” were applied. “Flow and timing” bias occurred when multiple clinical criteria were evaluated within the study, thus not applying the same reference standard. There was applicability concern in ≤ 1 domain in 64/66 studies (Fig. 3(B)). The principal applicability concern was introduced in the “patient selection” domain when only a narrow range of clinical diagnostic criteria were studied.

### Diagnostic performance of individual MRI descriptors for MD

**Fig. 4:**
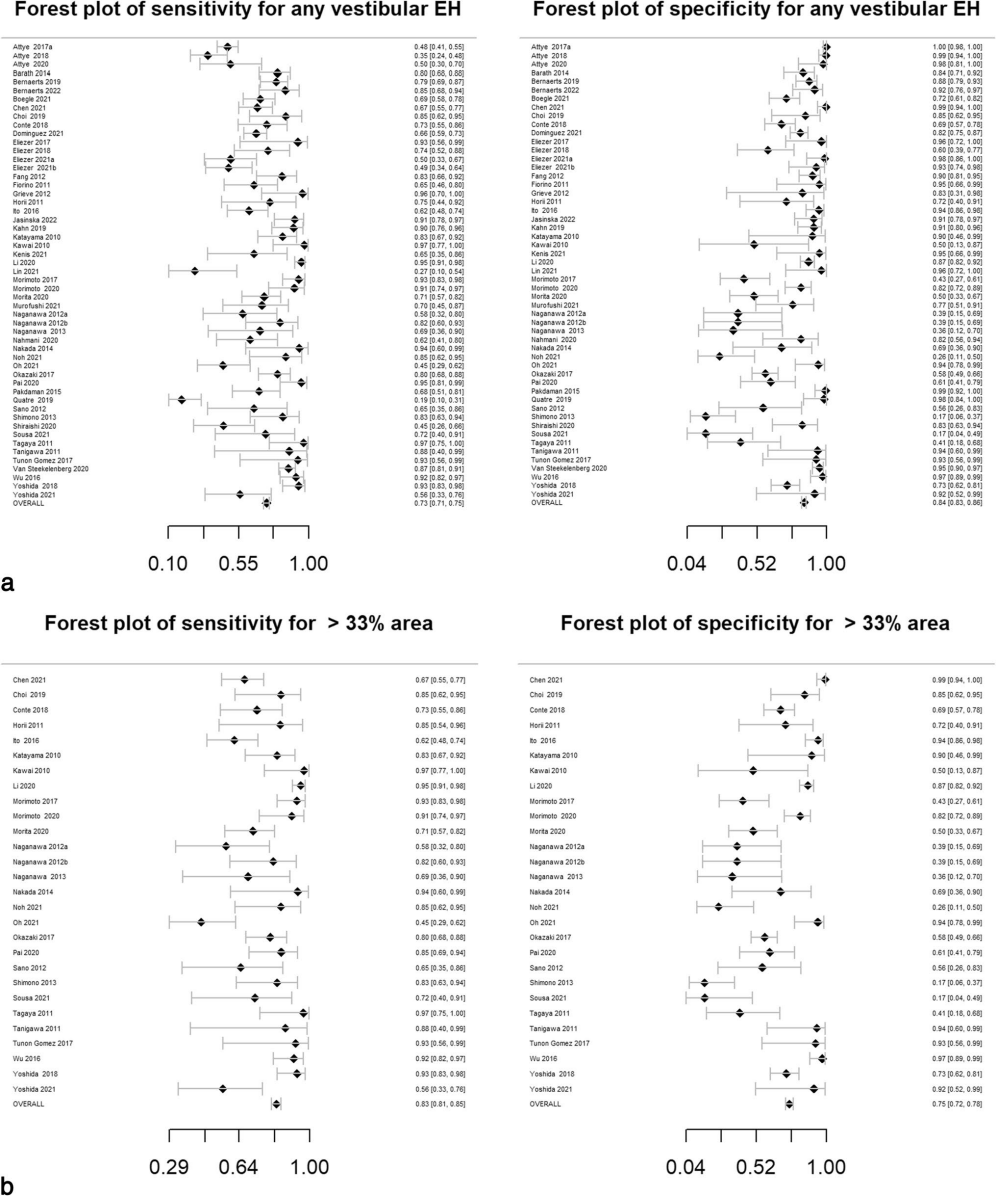

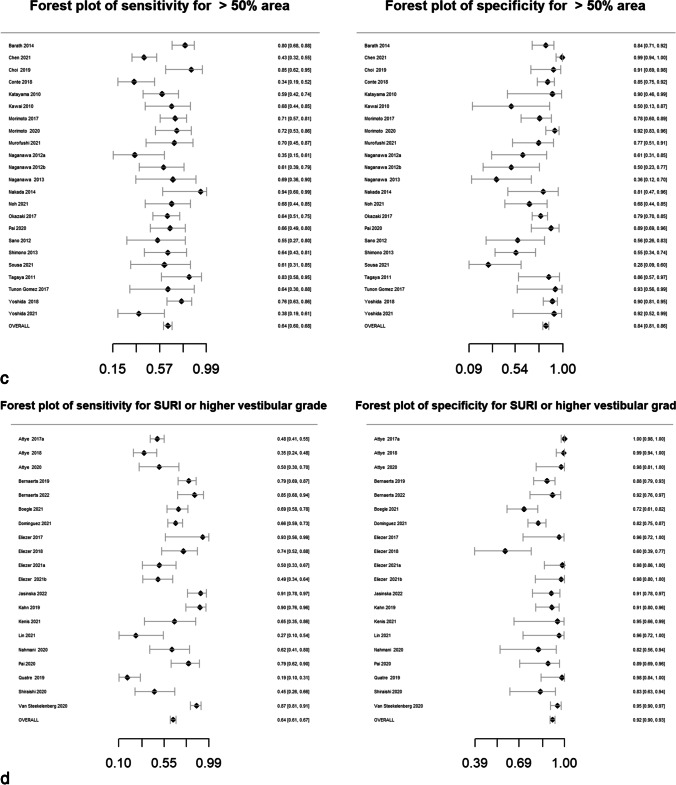

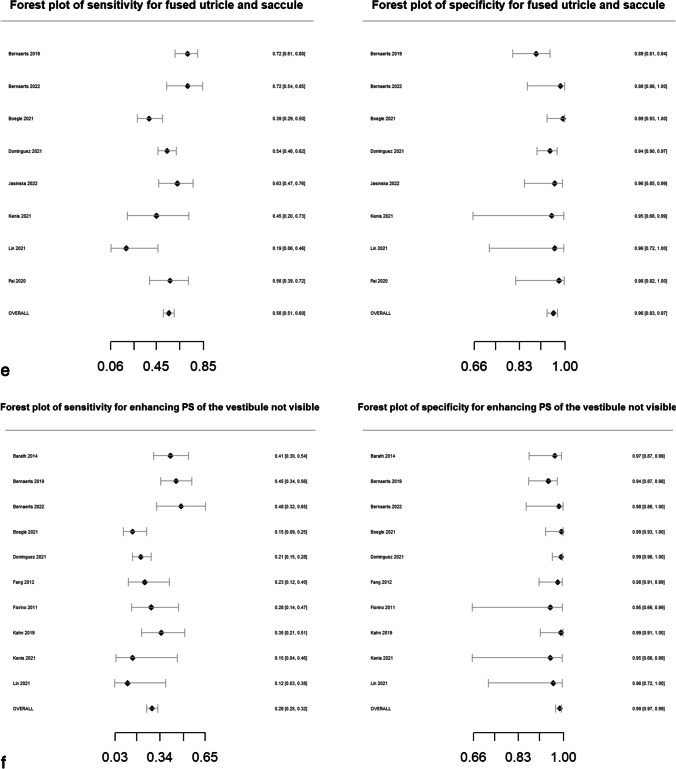

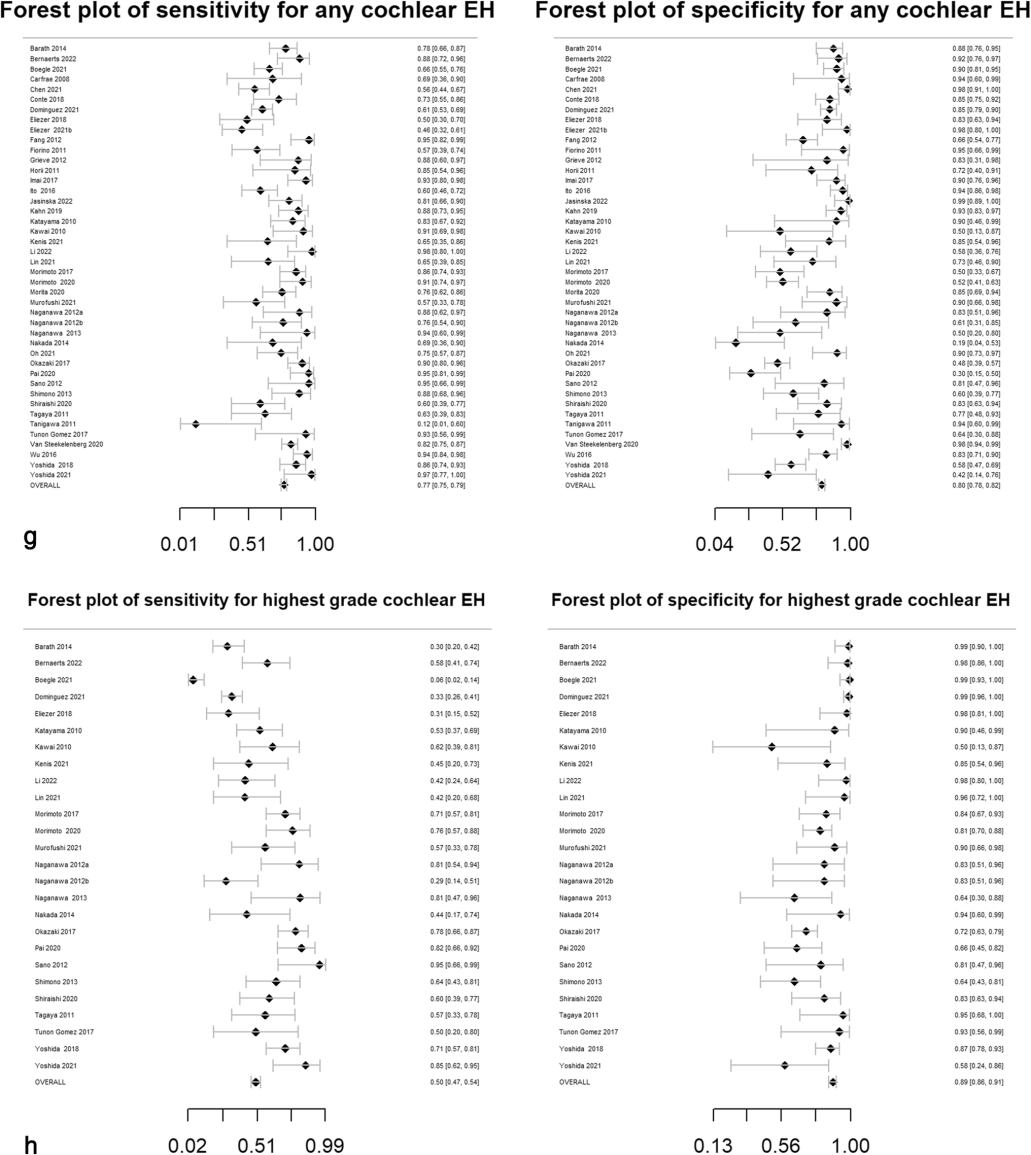

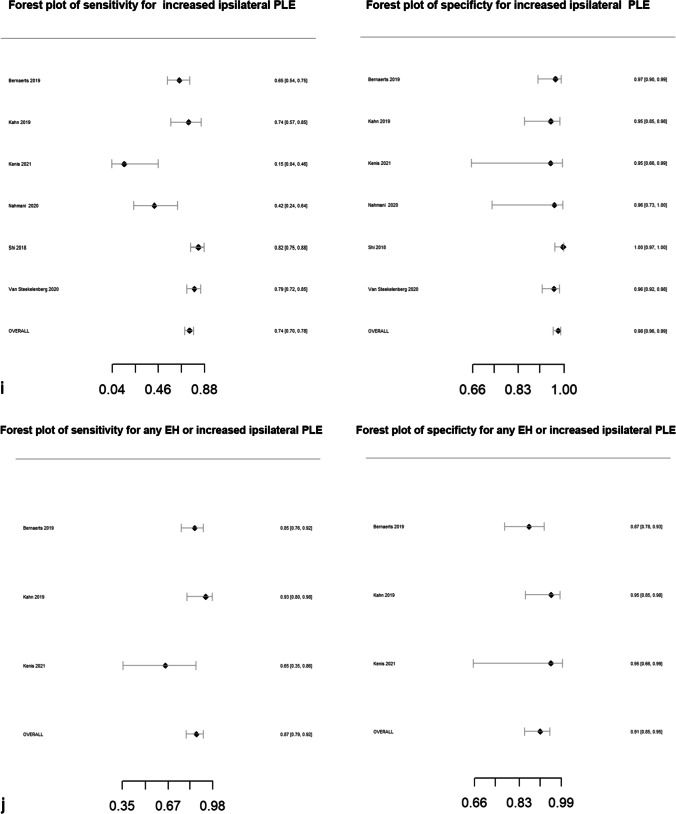

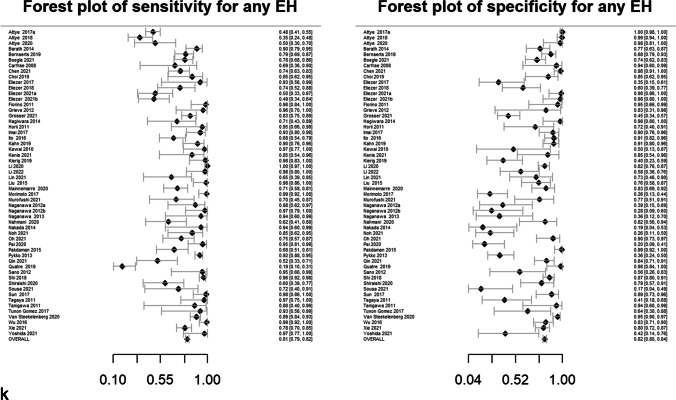
Forest plots with sensitivity and specificity for each MRI descriptor (**a**–**k**), incorporating all relevant reports and with pooled values

Pooled sensitivity, specificity, DOR, positive likelihood ratio and negative likelihood ratio are presented in Table 2. Forest plots for MRI descriptors are shown in Fig. 4, whilst summary ROC curves are shown in supplementary Fig. 1.

**Table 2:** Pooled sensitivity, specificity, diagnostic odds ratio (DOR), area under the curve (AUC) and likelihood ratios for MRI descriptors. Data in parentheses are 95% confidence interval MRI descriptors. DOR > 15 are highlighted in bold type

MRI descriptors	No. studies	Pooled sensitivity (%)	Pooled specificity (%)	Pooled diagnostic odds ratio (DOR)*	Pooled AUC	Pooled positive likelihood ratio*	Pooled negative likelihood ratio*
Any vestibular EH	53	73 (71, 75)	84 (83, 86)	14.6 (12.5, 16.6)	0.84	4.7 (4.2, 5.2)	0.32 (0.30, 0.34)
> 33% area of ES relative to total vestibular fluid area	28	83 (81, 85)	75 (72, 78)	14.7 (11.7, 18.5)	0.83	3.3 (3.0, 3.7)	0.23 (0.19, 0.26)
> 50% area of ES relative to total vestibular fluid area	23	64 (60, 68)	84 (81, 86)	9.3 (7.1, 12.2)	0.75	4.0 (3.3, 4.8)	0.42 (0.38, 0.48)
SURI or higher vestibular grade	**20**	**64 (61, 67)**	**92 (90, 93)**	**19.9 (15.4, 25.6)**	**0.88**	**7.8 (6.3, 9.6)**	**0.39 (0.36, 0.43)**
Fused utricle and saccule	**8**	**56 (51, 60)**	**96 (93, 97)**	**27.8 (16.6, 46.5)**	**0.89**	**12.9 (8.1, 20.5)**	**0.46 (0.42, 0.52)**
Enhancing PS of the vestibule not visible	**10**	**28 (25, 32)**	**99 (97, 99)**	**26.9 (12.8, 56.8)**	**0.87**	**19.6 (9.5, 40.3)**	**0.73 (0.69, 0.77)**
Any cochlear EH	43	77 (75, 79)	80 (78, 82)	13.2 (11.1, 15.8)	0.86	3.8 (3.4, 4.2)	0.29 (0.26, 0.32)
Highest grade cochlear EH	26	50 (47, 54)	89 (86, 91)	8.0 (6.1, 10.4)	0.81	4.5 (3.6, 5.5)	0.56 (0.52, 0.61)
Increased ipsilateral PLE	**6**	**74 (70, 78)**	**98 (96, 99)**	**131.7 (66.9, 259.2)**	**0.96**	**34.4 (18.3, 64.7)**	**0.26 (0.22, 0.31)**
Increased ipsilateral PLE or any EH	**3**	**87 (79, 92)**	**91 (85, 95)**	**64.8 (29.7, 141.2)**	**0.94**	**9.6 (5.6, 16.3)**	**0.15 (0.09, 0.23)**
Any EH	**55**	**81 (79, 82)**	**82 (80, 84)**	**18.9 (16.3, 22.0)**	**0.88**	**4.5 (4.1, 4.9)**	**0.24 (0.22, 0.26)**

*Diagnostic odds ratio (DOR) = (sensitivity × specificity) / (1-sensitivity) × (1-specificity) and is a global measure of performance of the MRI descriptor in diagnosing MD. It is the ratio of the odds of demonstrating the MRI descriptor in ears with MD relative to the odds of demonstrating the MRI descriptor in an ear without MD

Positive likelihood ratio = sensitivity /(1-specificity) and is the probability of demonstrating the MRI descriptor in an ear with MD, compared to the probability of demonstrating the MRI descriptor in an ear without MD Negative likelihood ratio = (1-sensitivity)/specificity) and is the probability of not demonstrating the MRI descriptor in an ear with MD, compared to the probability of not demonstrating the MRI descriptor in an ear without MD Since likelihood ratios are based on a ratio of sensitivity and specificity, they do not vary in different populations or settings and can be applied at the individual patient level

All MRI descriptors were highly informative, with DORs ranging from 8.0 (6.1, 10.4) for “highest grade cochlear EH” to 131.7 (66.9, 259.2) for “increased ipsilateral PLE”. Five of the 11 MRI descriptors achieved a pooled specificity of > 90%: “SURI or higher vestibular grade” (92%; 95% CI: 90%, 93%), “fused utricle and saccule” (96%; 95% CI: 93%, 97%), “enhancing PS of the vestibule not visible” (99%; 95% CI: 97%, 99%), “increased ipsilateral PLE” (98%; 95% CI: 96%, 99%) and “increased ipsilateral PLE or any EH” (91%; 95% CI: 85%, 95%). Of these, the highest sensitivity was achieved with “increased ipsilateral PLE or any EH” (87%; 95% CI: 79%, 92%) which demonstrated a pooled DOR of 64.8 (95% CI: 29.7%, 141.2%). The other MRI descriptors with a sensitivity greater than 80% were “ > 33% area of ES relative to total vestibular fluid area” (83%; 95% CI: 81%, 85%) and “any EH” (81%; 95% CI: 79%, 82%).

### Heterogeneity

All MRI descriptors demonstrated heterogeneity of sensitivity (Cochran’s *Q* test, *p* < 0.001). There were 4/11 MRI descriptors judged to show consistent specificity; however, 7/11 were heterogeneous predictors (Cochran’s *Q* test, *p* < 0.001) (supplementary 8). This heterogeneity is also reflected in the forest plots (Fig. 4) and Deek’s funnel plots (supplementary Fig. 2).

### Clinical classifications and other covariates

The results of subgroup analysis for the clinical classifications and the meta-regression for other co-variates are shown in Tables 3 and 4. When “definite 2015” MD classification was used, “increased ipsilateral PLE or any EH” achieved improved sensitivity of 89% (95% CI: 83%, 95%) and specificity of 91% (95% CI: 86%, 96%). There was no significant difference in diagnostic performance for any MRI descriptors between “definite 2015” and either “probable 2015” or “definite 1995” clinical classifications.

**Table 3:** Subgroup analysis for diagnostic performance of MRI descriptors in definite 2015, cMD and vMD

MRI descriptor	Clinical classification	No. of studies	*p* value (chi-sq) for each clinical subgroup v definite 2015*	Sensitivity (%)	Specificity (%)	Diagnostic odds ratio (DOR)
**Any vestibular EH**						
	Definite 2015	24	NA	76 (73, 78)	86 (84, 87)	18.56 (15.23, 22.62)
	Cochlear hydrops (cMD)	10	**.002**	51 (42, 61)	74 (69, 79)	3.02 (1.09, 10.33)
	Vestibular hydrops (vMD)	7	**.003**	40 (31, 50)	82 (78, 87)	3.15 (1.91, 5.21)
** > 33% area of ES relative to total vestibular fluid area**						
	Definite 2015	10	NA	86 (82, 89)	77 (74, 81)	20.09 (14.36, 28.12)
	Cochlear hydrops (cMD)	8	**.049**	72 (60, 75)	42 (30, 54)	1.91 (0.85, 4.29)
	Vestibular hydrops (vMD)	4	.20	71 (65, 88)	55 (41, 69)	3.07 (1.13, 8.30)
** > 50% area of ES relative to total vestibular fluid area**						
	Definite 2015	8	NA	55 (48, 61)	87 (83, 90)	7.95 (5.34, 11.84)
	Cochlear hydrops (cMD)	8	**.02**	45 (30, 59)	33 (19, 46)	1.65 (0.76, 3.60)
**SURI or higher vestibular grade**						
	Definite 2015	15	NA	71 (67, 74)	91 (90, 93)	25.53 (19.29, 33.79)
	Vestibular hydrops (vMD)	3	**.008**	28 (18, 39)	88 (84, 92)	2.98 (1.56, 5.68)
**Fused utricle and saccule**						
	Definite 2015	7	NA	68 (63, 74)	96 (94, 98)	46.73 (27.08, 80.63)
**Enhancing PS of the vestibule not visible**						
	Definite 2015	6	NA	35 (30, 41)	99 (98, 100)	41.11 (16.43, 102.93)
**Any cochlear EH**						
	Definite 2015	18	NA	79 (76, 82)	82 (79, 84)	16.64 (13.00, 21.31)
	Cochlear hydrops (cMD)	8	**.04**	61 (51, 71)	81 (76, 86)	6.69 (3.85, 11.63)
	Vestibular hydrops (vMD)	6	**.002**	40 (30, 51)	84 (79, 89)	3.54 (2.00, 6.26)
**Highest grade cochlear EH**						
	Definite 2015	10	NA	55 (49, 61)	88 (86, 91)	9.24 (6.45, 13.22)
	Cochlear hydrops (cMD)	9	.30	41 (30, 52)	94 (90, 97)	10.12 (4.97, 20.58)
	Vestibular hydrops (vMD)	4	.30	22 (10, 34)	99 (97, 100)	23.33 (4.90, 111.07)
**Asymmetric ipsilateral PLE**						
	Definite 2015	6		77 (73, 81)	98 (97, 99)	167.79 (83.60, 336.75)
**Asymmetric ipsilateral PLE or any EH**						
	Definite 2015	3		89 (83, 95)	91 (86, 96)	80.67 (35.27, 184.48)
**Any EH**						
	Definite 2015	22	NA	80 (78, 82)	85 (83, 87)	22.40 (18.30, 27.41)
	Cochlear hydrops (cMD)	8	.09	69 (58, 80)	79 (72, 85)	8.37 (4.34, 16.12)
	Vestibular hydrops (vMD)	4	**.007**	20 (9, 32)	87 (80, 94)	1.75 (0.70, 4.40)

The subgroup summary indices are calculated based on the presumption of homogeneity within each subgroup

^*^Clinical classifications are only tabulated when the MRI descriptor is evaluated in 3 or more studies with n > 5 MD ear cohort; *p* < 0.05 in bold type

**Table 4:** *p* values and significant co-variates on meta-regression

MRI descriptors*	**Control group type**	**Route of gadolinium administration:** IV v IT	**Number of image reviewers:** single v multiple observers	**Analysed:** ear basis v patient basis	**Bone signal:** Intermediate v low	**Risk of bias and applicability:** low risk of bias (any domain v none)/ high applicability (any v none)	**Study design:** prospective v other/consecutive recruitment v other
Any vestibular EH	.49	**.005**	**.03**	.26	**.003**	**.026/.026**	.08/.88
> 33% area of ES relative to total vestibular fluid area	.20	** < .001**	.11	.48	.44	**.03/**.09	.09/.72
> 50% area of ES relative to total vestibular fluid area	.824	.99	.07	.82	.70	.11/**.02**	.61/.69
SURI or higher vestibular EH grade	NA	NA	.77	NA	.14	.09/.7	**.005/**NA
Fused utricle and saccule	NA	NA	.63	NA	NA	NA/.77	NA/.64
Any cochlear EH	.20	.20	.18	.174	**.02**	.20/.45	.41/.14
Highest grade cochlear EH	.30	.66	.13	.30	**.02**	.48/.44	.41/.71
Any EH	.82	**.04**	**.01**	.98	**.03**	.11/.46	.24/.78

NA: subgroups with fewer than 3 eligible studies are not tabulated

^*^Eight MRI descriptors are tabulated. Meta-regression results for the MRI descriptors “Enhancing PS of the vestibule not visible”, “Increased ipsilateral PLE” and “Increased ipsilateral PLE or any EH” are not tabulated since subgroups included fewer than 3 eligible studies for all the co-variates

***p* < .05 in bold type**

With respect to the monosymptomatic classifications, the diagnostic performance of “high grade cochlear EH” (sensitivity 41%, specificity 94%, DOR 10.12) and “any EH” descriptors (sensitivity 69%, specificity 79%, DOR 8.37) did not significantly differ between “cMD” and “definite 2015” MD ears (*p* = 0.3; *p* = 0.09). As for vMD, the MRI descriptors “any EH” (sensitivity 20%, specificity 87%, DOR 1.75), “any vestibular EH” (sensitivity 40%, specificity 82%, DOR 3.15) and “any cochlear EH” (sensitivity 40%, specificity 84%, DOR 3.54) demonstrated low sensitivity and the diagnostic performance was inferior to “definite 2015” MD.

The meta-regression showed that the type of the control group type had no significant influence on the diagnostic performance. Regarding other covariates, “any EH” and “any vestibular EH” showed superior diagnostic performance with multiple observers or intra-tympanic gadolinium administration. Superior diagnostic performance was achieved with sequences or post-processing which depicted bone as intermediate signal for four MRI descriptors (Table 4).

The Deek’s funnel plots demonstrated a small studies effect (*p* < 0.05) for three MRI descriptors.

## Discussion

Despite increasing clinical application and impact on the diagnostic paradigm of Meniere’s disease (MD), there remains inconsistency in how delayed post-gadolinium MRI is interpreted and applied in clinical settings. This systematic review and meta-analysis evaluated 11 MRI descriptors for their ability to distinguish MD ears as defined by various clinical criteria. All descriptors were considered highly informative with DORs ranging from 8.0 (6.1, 10.4) to 131.7 (66.9, 259.2). “Increased ipsilateral perilymphatic enhancement (PLE)”, alone or in combination with “any endolymphatic hydrops (EH)”, demonstrated the highest DORs. This combination achieved the highest sensitivity (87% (95% CI: 79.92%)) whilst maintaining high specificity (91% (95% CI: 85.95%)) for MD, although it was only evaluated in three studies. Evaluation of EH for MD diagnosis was best attained with MRI features assessing the endolymphatic space alone, rather than comparing it with the perilymphatic area. Such descriptors with high DORs of 19.9 (15.5, 25.6) and 27.8 (16.6, 46.5) were “saccule to utricle ratio inversion or higher vestibular grade” and “fused utricle and saccule”. Diagnostic performance did not differ across definite 2015, probable 2015 and definite 1995 clinical classifications for any MRI descriptor. “Highest grade cochlear EH” and “any EH” performed similarly in monosymptomatic cochlear MD to the clinical reference standard of “definite 2015” MD (*p* = 0.3; *p* = 0.09). Sequences or post-processing which depicted bone as intermediate signal demonstrated superior diagnostic performance for four MRI descriptors. High risk of bias and heterogeneity was noted across the eligible studies included in the meta-analysis.

The current study differs in several respects from previous systematic reviews of delayed post-gadolinium MRI in MD [89–93]. Firstly, our contemporary literature search resulted in 72 eligible studies compared with 11–43 studies in previous reviews, providing sufficient data to enable a meta-analysis and pooled statistics for the first time. Secondly, this review evaluated 11 MRI descriptors compared with 1–4 descriptors in prior publications. Finally, inclusion and subgroup analysis of all clinical classifications explored the diagnostic performance of MRI in different symptomatic presentations.

The appropriate selection of specific versus sensitive MRI descriptors for the diagnosis of MD may depend on the clinical setting, as illustrated by a comparison of different vestibular EH descriptors. For instance, when low risk treatment or non-destructive interventions (e.g., intratympanic steroids) are being considered then overdiagnosis may be acceptable so “ > 33%” area of endolymph relative to total fluid area “would be a reasonable descriptor due to its higher pooled sensitivity (83%), despite low specificity (75%)”. Conversely, if vestibular-destructive procedures or trials of new interventions with potential morbidity are envisaged, then application of highly specific descriptors would be more appropriate. Regarding potential application to automated MRI analysis, it is of interest that vestibular MRI descriptors evaluating endolymphatic appearances alone demonstrated superior diagnostic performance, since current techniques focus on comparison with the perilymphatic space area.

Evaluation of MRI descriptors across the whole range of symptomatic presentations provided evidence for their diagnostic performance in differing clinical phenotypes [8]. The meta-regression indicated that most descriptors had a similar ability to diagnose MD when applying the current “definite 2015” reference standard or alternative clinical classifications, supporting the role of MRI in wider clinical situations. This extended to monosymptomatic presentations for some descriptors, with the presence of “any EH” being able to detect cMD ears with DOR of 8.37 (4.34, 16.12).

There are limitations to the current study, with respect to both the review process and the evidence available. Firstly, although the risk of missing data was minimised as far as possible, a body of non-English language literature (36 screened studies) was not reviewed. Secondly, since only a limited range of MRI descriptors were applied in individual studies, it was not feasible to perform head-to-head comparisons, introducing bias due to the indirect comparison of individual MRI descriptors. Thirdly, as the eligible studies principally focused on definite MD, the subgroup analysis of atypical forms of MD for less frequently analysed MRI descriptors yielded insufficient numbers for pooling data. Fourthly, it would have been pertinent to perform meta-regression for the “Increased ipsilateral PLE” descriptor with respect to the control group of “other audio vestibular disorders” (since it may also occur with differential diagnoses such as perilymphatic fistula) and for constant versus variable flip angle FLAIR sequences (since this influences the degree of PLE); however, this was precluded due to the limited number of eligible studies. Fifthly, variations in sensitivity (all descriptors) and specificity (7/11 descriptors) led to significant heterogeneity across studies. Meta-regression demonstrated that this was at least partly due to variable clinical classifications, MRI technique, analysis, study design, applicability and bias. Finally, the high level of bias should be considered. In particular, all eligible studies were case controlled, potentially resulting in an overestimation of diagnostic accuracy [105].

## Conclusion

This systematic review and meta-analysis evaluated the relative performance of MRI descriptors for the diagnosis of MD. “Increased ipsilateral PLE” was a key descriptor, and in combination with EH, it achieved optimal specificity with sensitivity. MRI descriptors of EH which did not rely on a comparison with perilymphatic area showed the best diagnostic performance for MD. MRI diagnosis of EH can be usefully applied across a range of clinical classifications including monosymptomatic cMD. Future research and meta-analysis would benefit from consensus on standardised MRI descriptors and a minimum clinical data set, whilst MRI descriptors should also be evaluated for prognosis and prediction of treatment response through longitudinal studies.

### Supplementary Information

Below is the link to the electronic supplementary material.

Supplementary file1 (PDF 220 KB)

Supplementary file2 (PDF 560 KB)

Supplementary file3 (PDF 203 KB)

Supplementary file4 (PDF 160 KB)

Supplementary file5 (PDF 200 KB)

Supplementary file6 (PDF 292 KB)

Supplementary file7 (PDF 158 KB)

Supplementary file8 (PDF 257 KB)

Supplementary file9 (PDF 118 KB)

Supplementary file10 (PDF 109 KB)

## Data Availability

The data supporting this article is openly available from the King’s College London research data repository, KORDS, at 10.18742/20522376.

## Abbreviations

cMD: Cochlear Meniere’s disease
DOR: Diagnostic odds ratio
EH: Endolymphatic hydrops
ES: Endolymphatic space
MD: Meniere’s disease
PLE: Perilymphatic enhancement
PS: Perilymphatic space
SURI: Saccule to utricle ratio
vMD: Vestibular Meniere’s disease

## Declarations

### Guarantor

The scientific guarantor of this publication is Dr. SEJ Connor.

### Statistics and biometry

Mariusz T. Grzeda MSc and Babak Jamshidi PhD kindly provided statistical advice for this manuscript.

### Study subjects or cohorts overlap

Study subjects are acquired from literature search of previously reported cohorts.

### Methodology

• Methodology of reviewed literature includes prospective or retrospective case–control studies.
