# Supplementary material for: Delayed post gadolinium MRI descriptors for Meniere’s disease: a systematic review and meta-analysis
Source: Eur Radiol. 2023 May 12;33(10):7113–35. doi: 10.1007/s00330-023-09651-8 (PMC10511628; doi:10.1007/s00330-023-09651-8)
Supplement: Supplementary file 1 — Supplementary file1 (PDF 220 KB) [file 330_2023_9651_MOESM1_ESM.pdf]

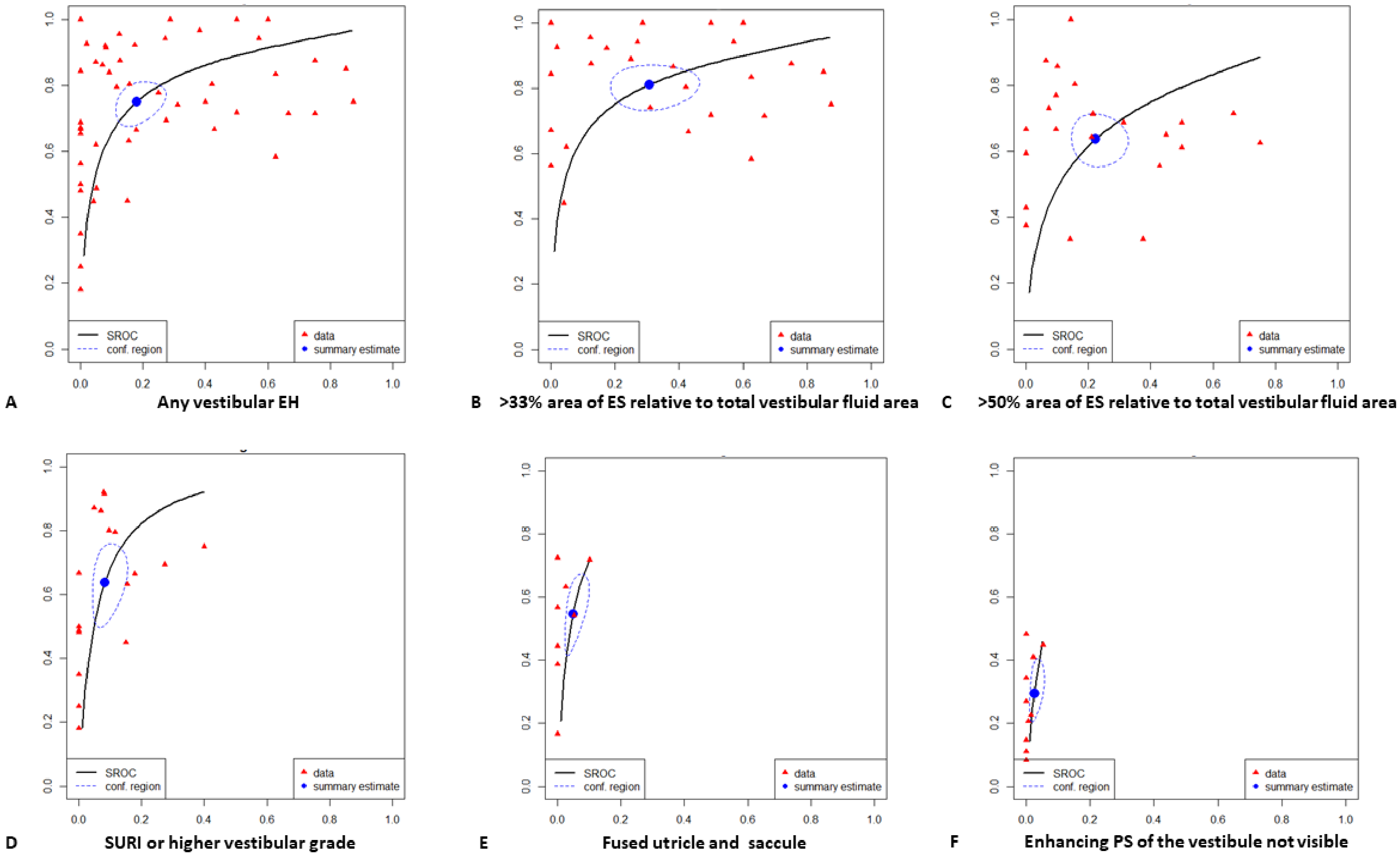
SROC
conf. region
data
summary estimate
0.0
0.2
0.4
0.6
0.8
1.0
A
Any vestibular EH
B
>33% area of ES relative to total vestibular fluid area
C
>50% area of ES relative to total vestibular fluid area
D
SURI or higher vestibular grade
E
Fused utricle and saccule
F
Enhancing PS of the vestibule not visible

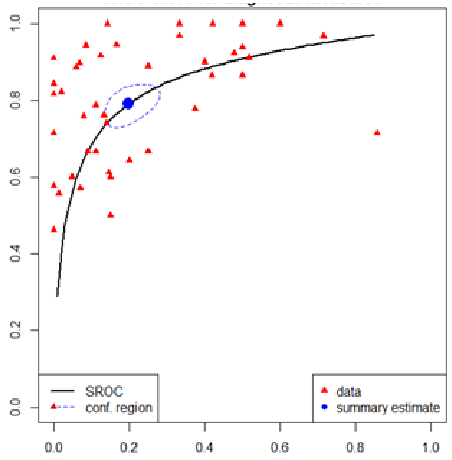

G Any cochlear EH

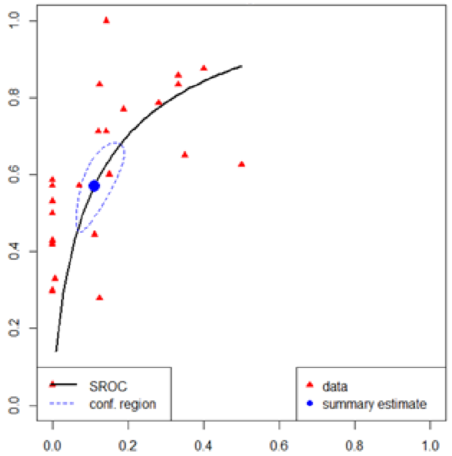

H Highest grade cochlear EH

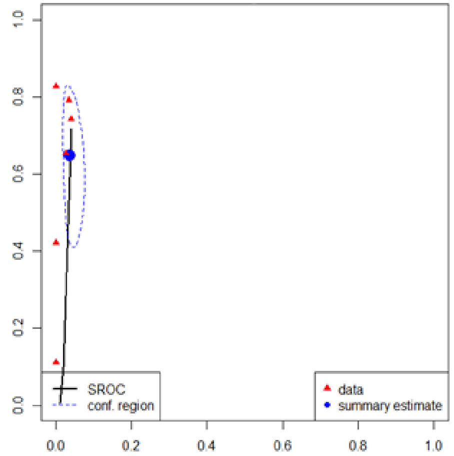

I Increased ipsilateral PLE

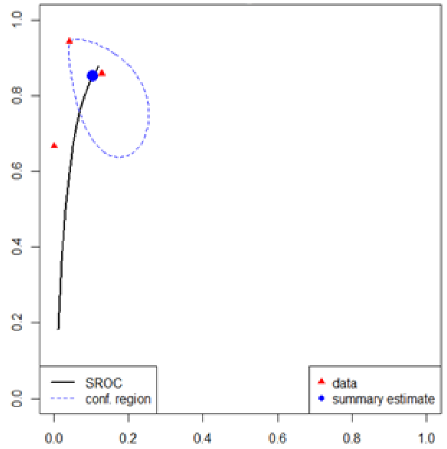

J Increased ipsilateral PLE or any EH

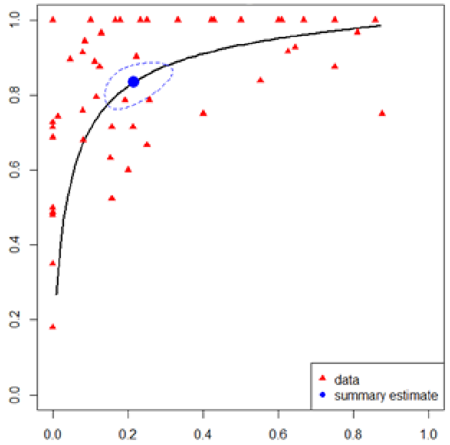

K Any EH

Supplementary figure 1: Summary ROC (SROC) curves for MRI descriptors (A-K). They indicate the relationship between the true positive rate (TPR) and the false positive rate (FPR) of the MRI descriptors at various thresholds used to distinguish MD ears from control ears. The y axis corresponds to true positive rate and the x axis to false positive rate. Pooled area under the curve (AUC) is demonstrated in table 2. They can be characterized in terms of the overall diagnostic odds ratio and the magnitude of inter-study heterogeneity in the odds ratio. Increasing odds ratio will move the curve to the upper left corner with increased AUC. There was a AUC >0.9 for "increased ipsilateral PLE" and "increased ipsilateral PLE or any EH" (I,J) however there were only a small number of studies . Smoothed fitting of the SROC curves is achieved with a regression model.
