# Supplementary material for: Delayed post gadolinium MRI descriptors for Meniere’s disease: a systematic review and meta-analysis
Source: Eur Radiol. 2023 May 12;33(10):7113–35. doi: 10.1007/s00330-023-09651-8 (PMC10511628; doi:10.1007/s00330-023-09651-8)
Supplement: Supplementary file 2 — Supplementary file2 (PDF 560 KB) [file 330_2023_9651_MOESM2_ESM.pdf]

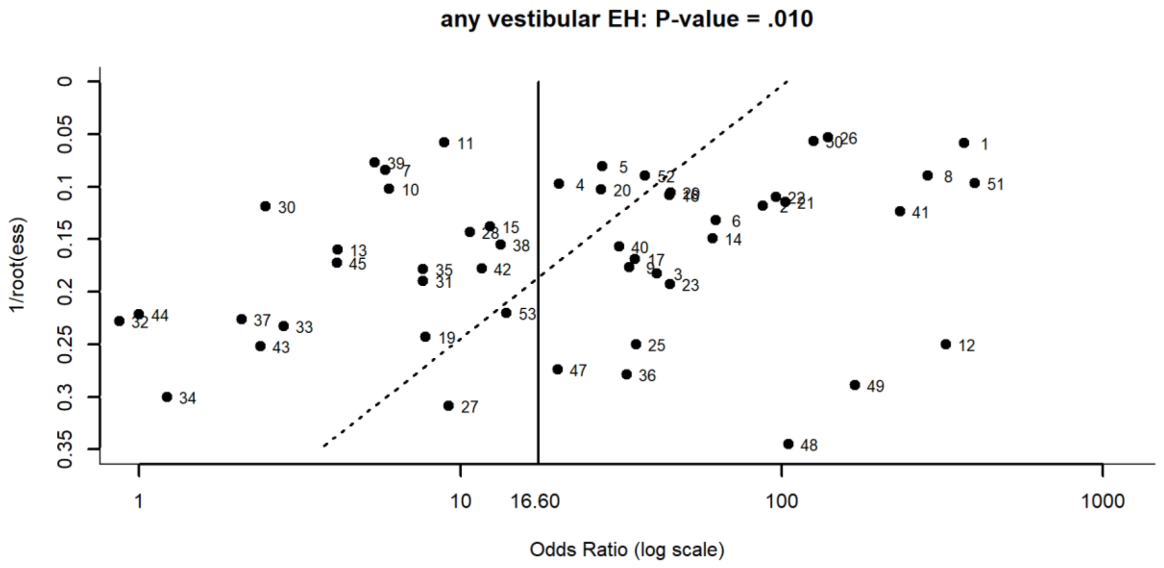
any vestibular EH: P-value = .010
1/root(ess)
0
0.05
0.1
0.15
0.2
0.25
0.3
0.35
1
10
16.60
100
1000
Odds Ratio (log scale)

A

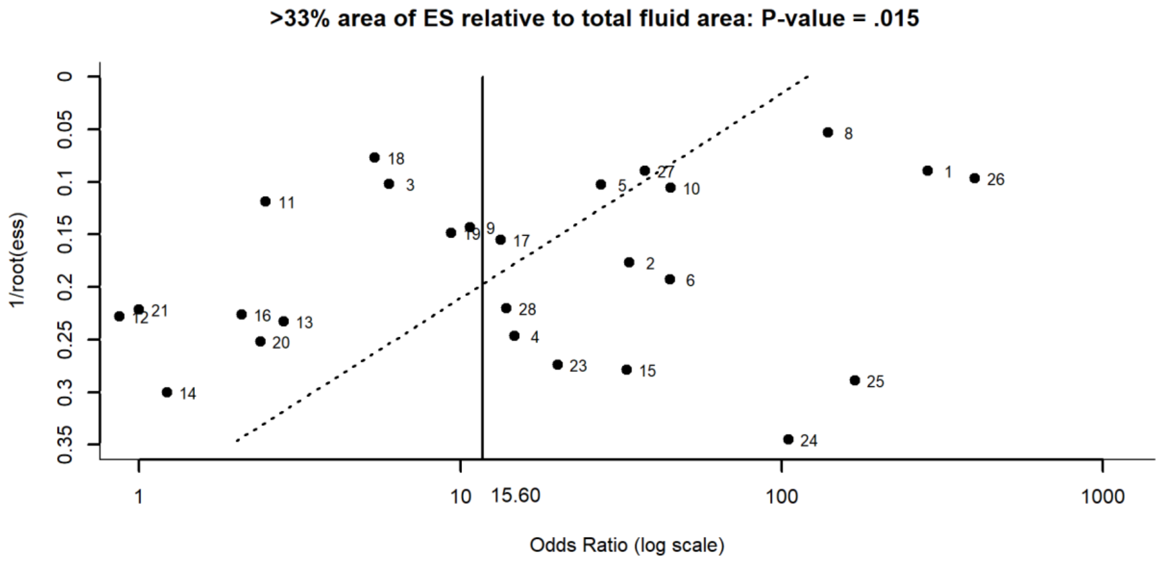
>33% area of ES relative to total fluid area: P-value = .015
1/root(ess)
0
0.05
0.1
0.15
0.2
0.25
0.3
0.35
1
10
15.60
100
1000
Odds Ratio (log scale)

B

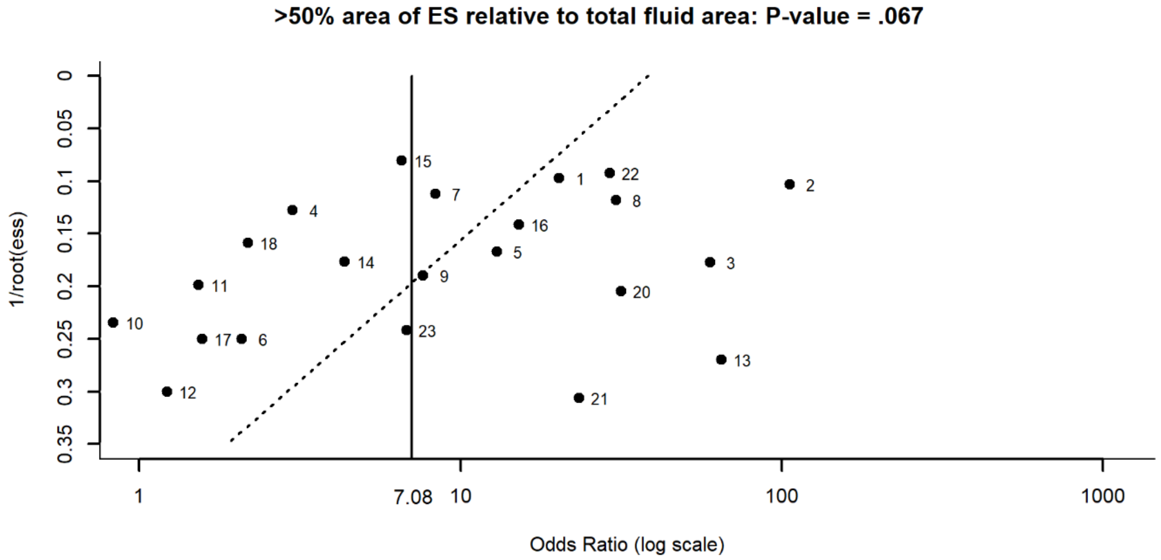
>50% area of ES relative to total fluid area: P-value = .067
1/root(ess)
0
0.05
0.1
0.15
0.2
0.25
0.3
0.35
1
7.08
10
100
1000
Odds Ratio (log scale)

C

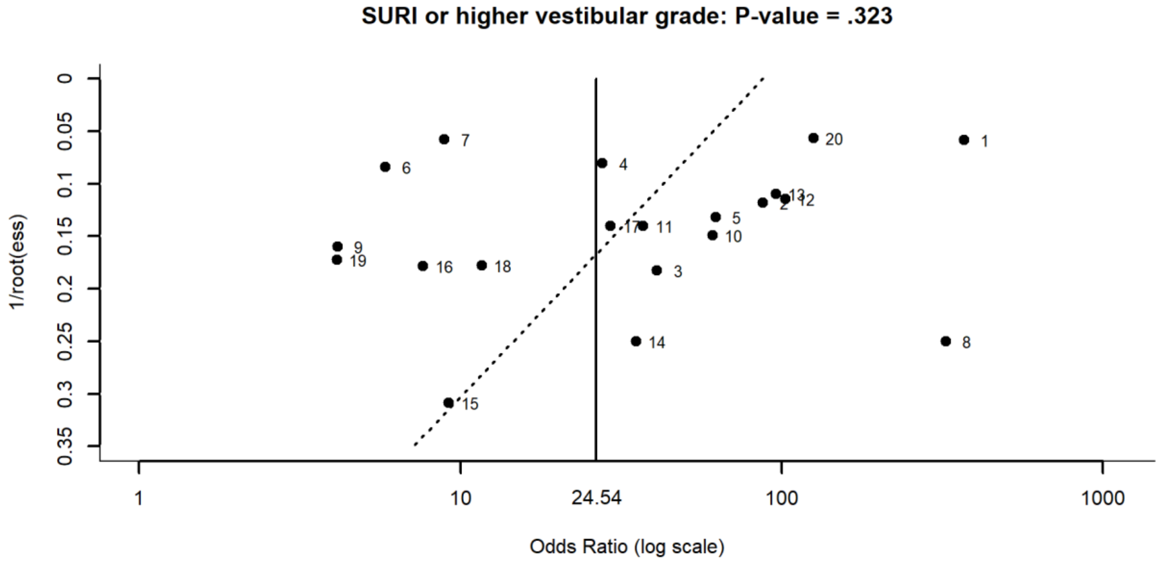
SURI or higher vestibular grade: P-value = .323
1/root(ess)
0
0.05
0.1
0.15
0.2
0.25
0.3
0.35
1
10
24.54
100
1000
Odds Ratio (log scale)

D

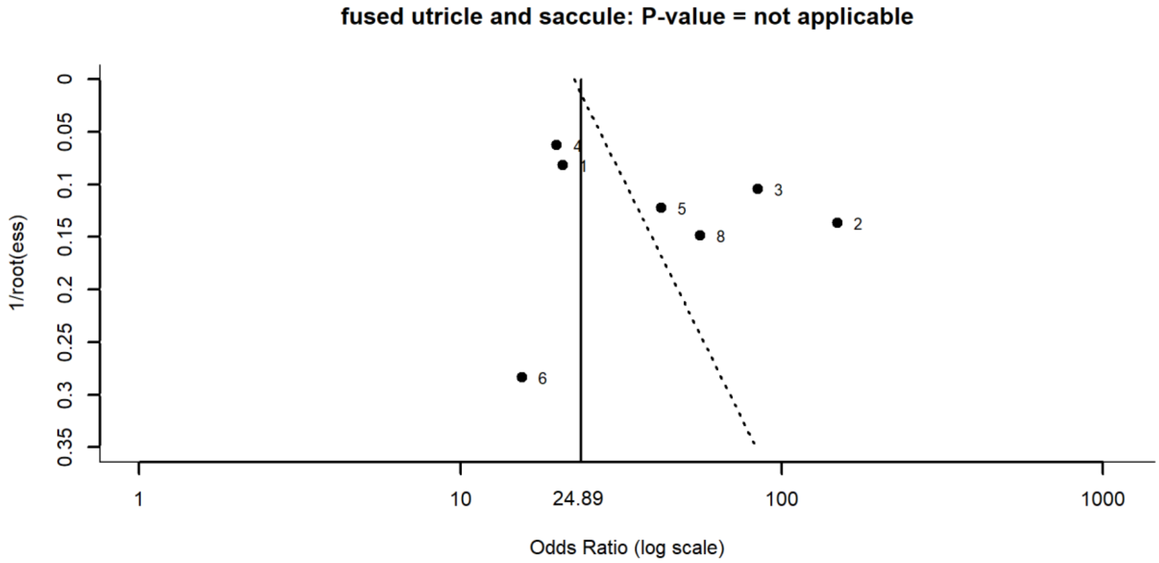
fused utricle and saccule: P-value = not applicable
1/root(ess)
0
0.05
0.1
0.15
0.2
0.25
0.3
0.35
4
1
3
5
2
8
6
1
10
24.89
100
1000
Odds Ratio (log scale)

E

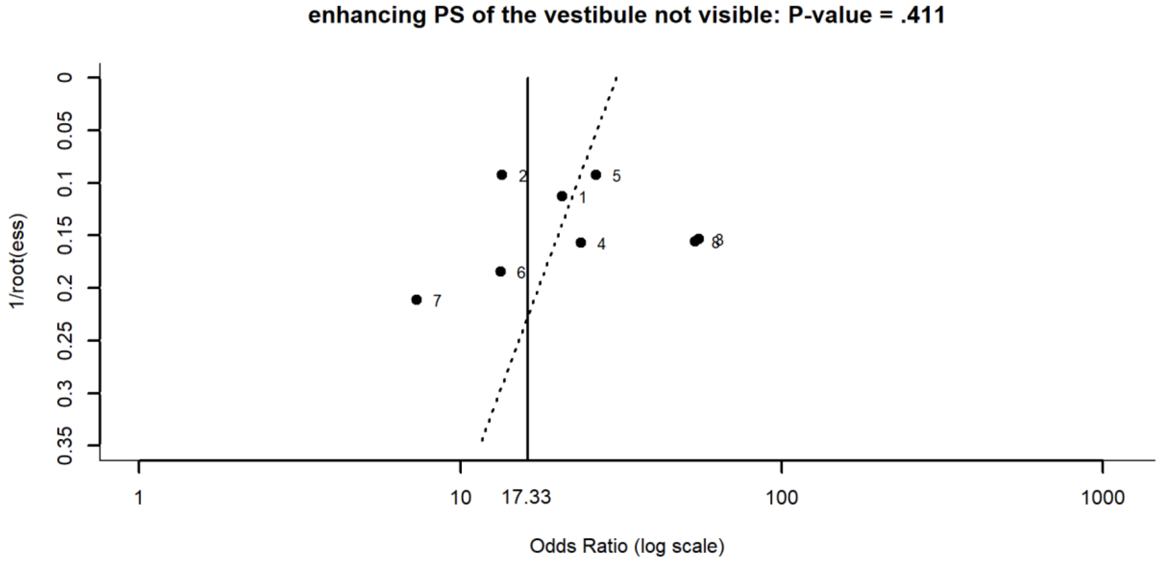
enhancing PS of the vestibule not visible: P-value = .411
1/root(ess)
0
0.05
0.1
0.15
0.2
0.25
0.3
0.35
2
5
1
4
6
7
1
10
17.33
100
1000
Odds Ratio (log scale)

G

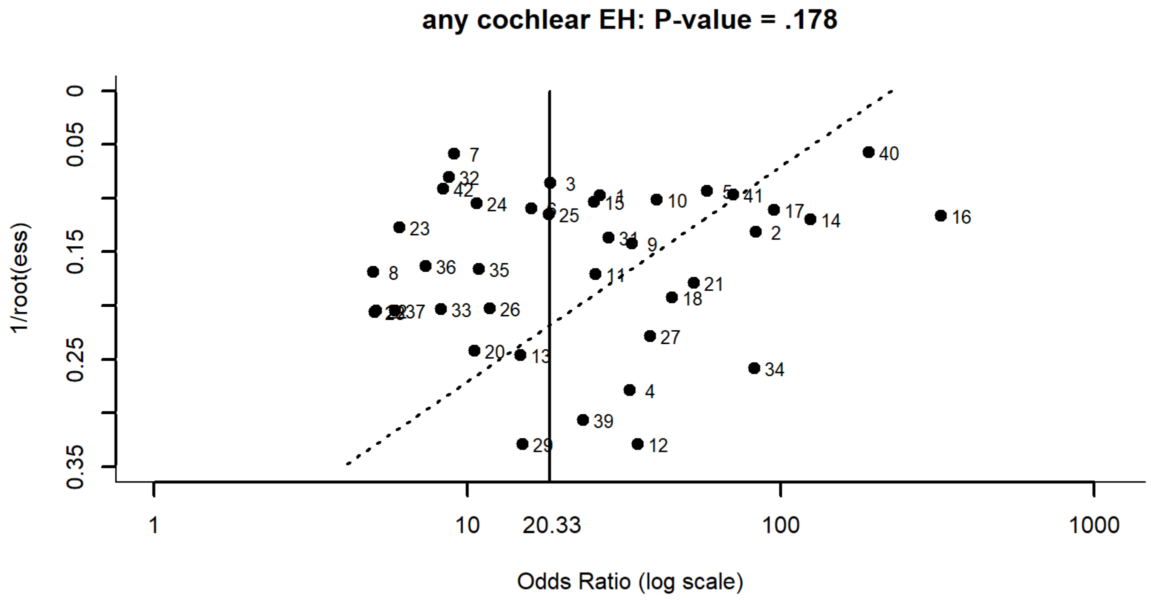
any cochlear EH: P-value = .178
1/root(ess)
0
0.05
0.15
0.25
0.35
1
10
20.33
100
1000
Odds Ratio (log scale)

F

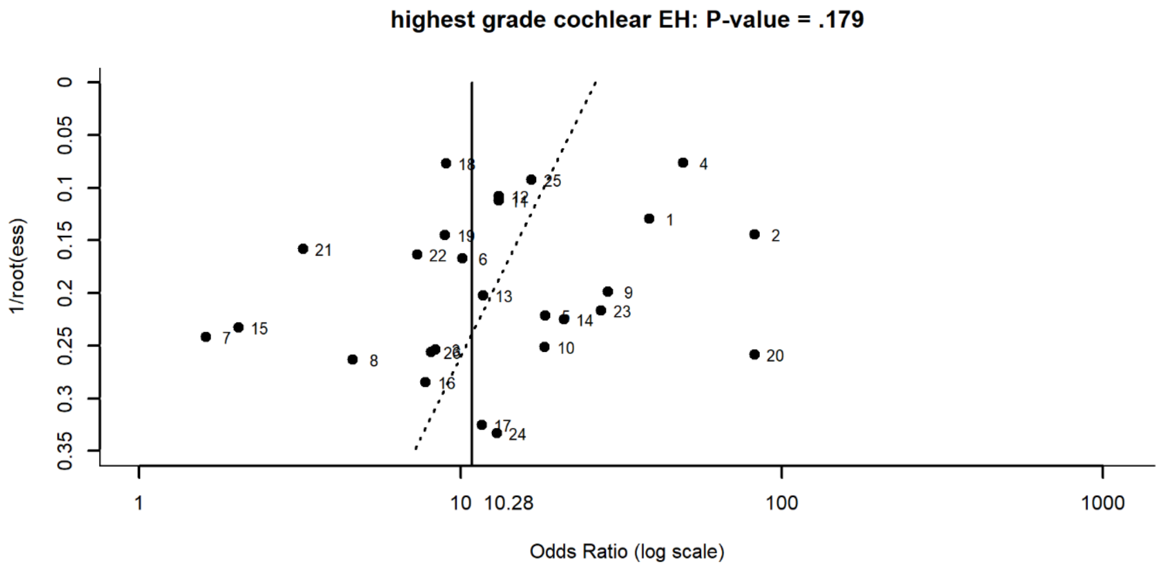
highest grade cochlear EH: P-value = .179
1/root(ess)
0
0.05
0.1
0.15
0.2
0.25
0.3
0.35
1
10
10.28
100
1000
Odds Ratio (log scale)

H

Any EH or increased ipsilateral PLE: P-value=not applicable

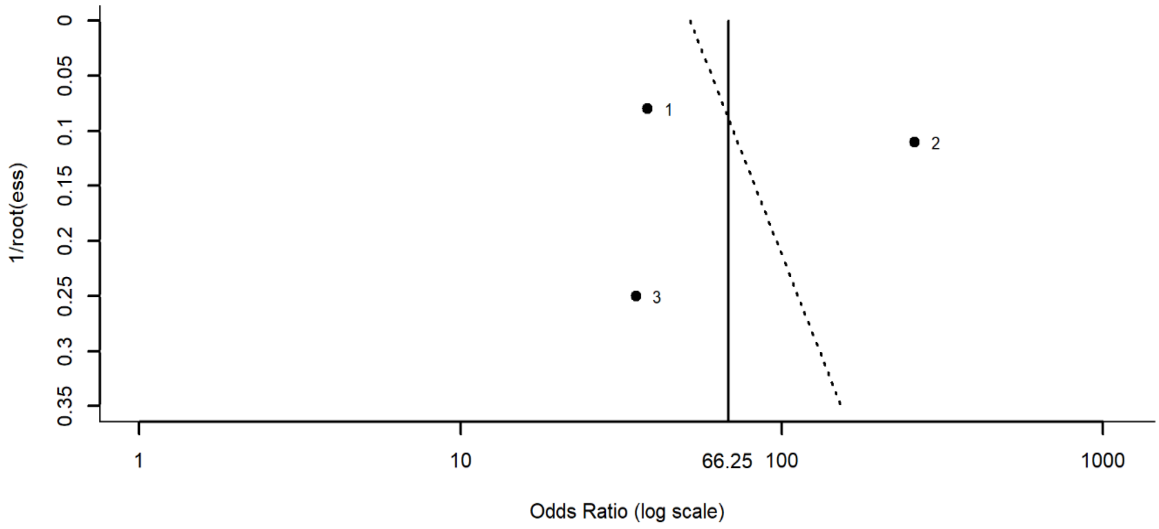

I

Increased ipsilateral PLE: P-value=not applicable

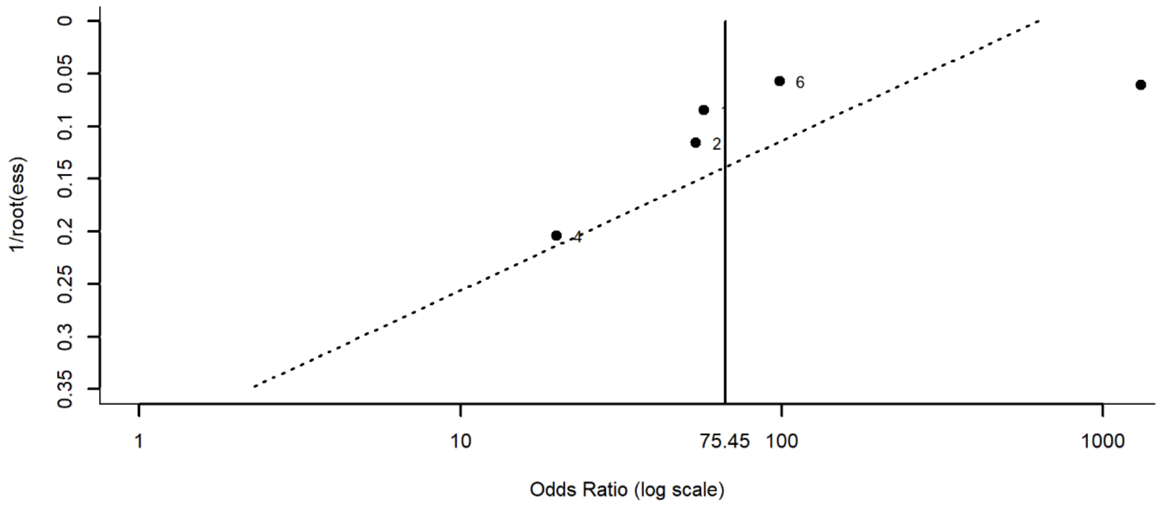

J

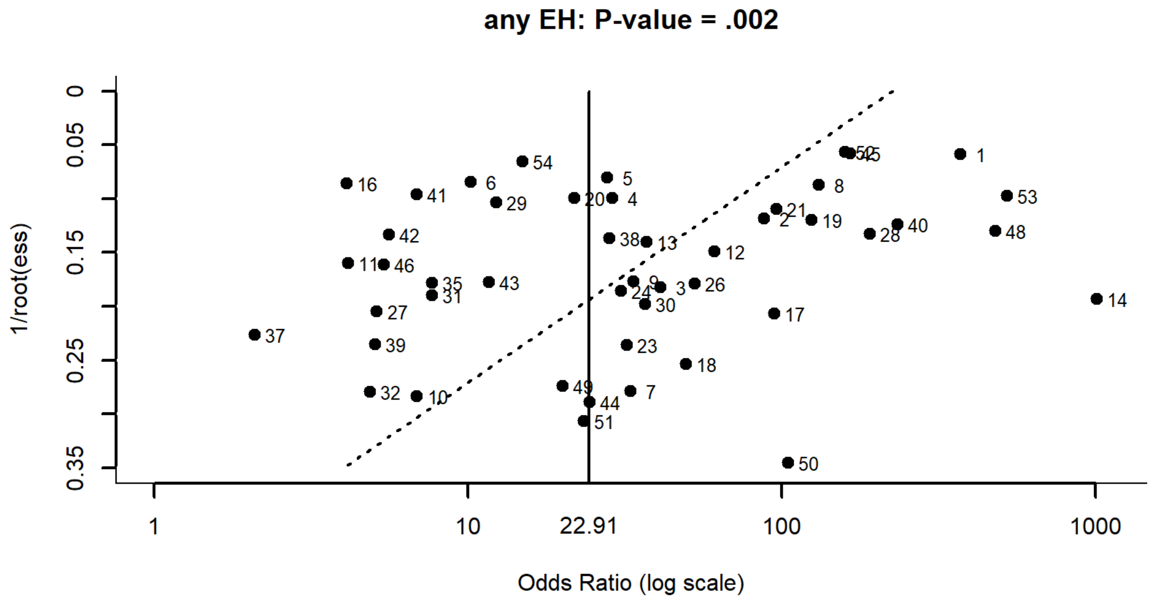

K

Supplementary figure 2: Deek's funnel plots for MRI descriptors applied to all eligible studies. These plots were used to evaluate publication bias and sample size effect for the 11 analysed MRI descriptors for eligible studies. Horizontal and vertical axes represent odds ratio and inverse of root of the effective sample size for different studies, and asymmetry indicates some source of bias or heterogeneity. A horizontal regression line would indicate absence of publication bias and sample size effect and reported P-values in plots belong to this symmetry test. It is of note that asymmetry implies bias, but symmetry does not necessarily mean that bias is absent. For three outcomes, due to the small number of studies, it was not possible to compute P-values however the plots are presented. It should be noted that these plots are based on the logarithmic scale of DORs with the figures representing the average DOR (obtained by calculating logarithm of DORs, then the average of log DORs, and finally exponential of the obtained average). The pooled DORs are demonstrated in table 2. Numbers correspond to the study numbers in the online data repository (see data availability).
