# Supplementary material for: Delayed post gadolinium MRI descriptors for Meniere’s disease: a systematic review and meta-analysis
Source: Eur Radiol. 2023 May 12;33(10):7113–35. doi: 10.1007/s00330-023-09651-8 (PMC10511628; doi:10.1007/s00330-023-09651-8)
Supplement: Supplementary file 3 — Supplementary file3 (PDF 203 KB) [file 330_2023_9651_MOESM3_ESM.pdf]

**<u>Database search terms</u>**<br><br>**Key words (Thesaurus/index and MESH terms) and free text search terms were adapted for each database search.**			
**Embase (last searched 17/2/22)**		**Medline (last searched 17/2/22)**	
#1	exp Ear Disease/	#1	exp Ear Diseases/
#2	exp dizziness/	#2	Dizziness/
#3	exp hearing disorder/	#3	exp Hearing Disorders/
#4	exp balance disorder/	#4	exp Ear/
#5	dizz*.mp.	#5	dizz*.mp.
#6	vert*.mp.	#6	vert*.mp.
#7	ear*.mp.	#7	ear*.mp.
#8	hear*.mp.	#8	hear*.mp.
#9	Meniere*.mp.	#9	meniere*.mp.
#10	exp Meniere disease/	#10	exp Meniere Disease/
#11	endolymph*.mp.	#11	endolymph*.mp.
#12	perilymph*.mp.	#12	perilymph*.mp.
#13	hydrop*.mp.	#13	hydrop*.mp.
#14	exp nuclear magnetic resonance/	#14	exp Endolymphatic Hydrops/
#15	magnetic resonance imag*.mp.	#15	exp Perilymph/
#16	MRI.mp.	#16	exp Endolymph/
#17	gadolinium/	#17	exp Magnetic Resonance Imaging/
#18	exp nuclear magnetic resonance imaging agent/	#18	MAGNETIC RESONANCE IMAG*.mp.
#19	GADOLINIUM.mp.	#19	MRI.mp.
#20	CONTRAST.mp.	#20	Gadolinium/
#21	GBCM.mp.	#21	Gadolinium DTPA/
#22	intratympanic.mp.	#22	gadolinium.mp.
#23	intra-tympanic.mp.	#23	CONTRAST.mp.
#24	#1 OR #2 OR #3 OR #4 OR #5 OR #6 OR #7 OR #8	#24	GBCM.mp.
#25	#9 OR #10 OR #11 OR #12 OR #13	#25	INTRATYMPANIC.mp.
#26	#14 OR #15 OR #16 OR #17 OR #18	#26	INTRA-TYMPANIC.mp.
#27	#19 OR #20 OR #21 OR #22 OR #23	#27	Injection, Intratympanic/
#28	#23 AND #24 AND #25 AND #26	#28	#1 OR #2 OR #3 OR #4 OR #5 OR #6 OR #7 OR #8

#29	limit 28 to yr="2000 -Current	#29	#9 OR #10 OR #11 OR #12 OR #13 OR #14 OR #15 OR #16
		#30	#17 OR #18 OR #19
		#31	#20 OR #21 OR #22 OR #23 OR #24 OR #25 OR #26 OR #27 OR #28
		#32	#28 AND #29 AND #30 AND #31
		#33	limit 32 to yr="2000 -Current"
**Web of Science and Scopus (last searched 17/2/22)**		**Cochrane Register of Controlled Trials (last searched 17/2/22)**	
#1	otologic*	#1	ear*
#2	SNHL	#2	hear*
#3	balance	#3	dizz*
#4	deaf*	#4	vert*
#5	dizz*	#5	MeSH descriptor: [Ear Diseases] explode all trees
#6	vert*	#6	MeSH descriptor: [Dizziness] explode all trees
#7	ear*	#7	MeSH descriptor: [Hearing Disorders] explode all trees
#8	hear*	#8	#1 OR #2 OR #3 OR #4 OR #5 OR #6 OR #6 OR #7
#9	tinnitus	#9	MeSH descriptor: [Meniere Disease] explode all trees
#10	audio	#10	MENIERE*
#11	aural	#11	MeSH descriptor: [Endolymphatic Hydrops] explode all trees
#12	Meniere*	#12	MeSH descriptor: [Perilymph] explode all trees
#13	labyrinth*	#13	MeSH descriptor: [Endolymph] explode all trees
#14	endolymph*	#14	ENDOLYMPH*
#15	perilymph*	#15	PERILYMPH*
#16	hydrop*	#16	HYDROP*
#17	cochle*	#17	#9 OR #10 OR #11 OR #12 0R #13 OR #14 OR #15 OR #16
#18	vestibul*	#18	MeSH descriptor: [Magnetic Resonance Imaging] explode all trees
#19	MR	#19	MR
#20	magnetic resonance imag*	#20	MRI
#21	MRI	#21	"MAGNETIC RESONANCE IMAG"
#22	gadolinium	#22	#18 OR #19 OR #20 OR #21
#23	contrast	#23	MeSH descriptor: [Gadolinium] this term only
#24	GBCM	#24	GADOLINIUM
#25	intra*tympanic	#25	CONTRAST
#26	#1 OR #2 OR #3 OR #4 OR #5 OR #6 OR #7 OR #8 OR #9 OR #10 OR #11	#26	GBCM
#27	#12 OR #13 OR #14 OR #15 OR #16 OR #17 OR #18	#27	INTRATYMPANIC
#28	#19 OR #20 OR #21	#28	INTRA-TYMPANIC
#29	#22 OR #23 OR #24 OR #25	#29	MeSH descriptor: [Injection, Intratympanic] this term only
#30	#26 AND #27 AND #28 AND #29	#30	#23 OR #24 OR #25 OR #26 OR #27 OR #28 OR #29
#31	#30 AND PUBYEAR >19	#31	#8 AND #17 AND #22 AND #30

**Hand searching/Reference harvesting/Grey literature**
**1) The five most frequently cited journals from the Scopus search were identified to be:**
Otology Neurotology<br>*Acta Oto-Laryngologica*<br>*The Laryngoscope*<br>*European Archives of Oto-rhino-laryngology*<br>*Journal of Laryngology and Otology*
The table of contents in these journals were hand searched from 2010-2021.
**2) Preprint documents, theses and grey literature was searched with the terms "ear "and "MRI"**<br>https://www.cadth.ca/resources/finding-evidence/grey-matters<br>https://www.cadth.ca/grey-matters-practical-tool-searching-health-related-grey-literature<br>https://ethos.bl.uk/Home.do<br>http://www.greynet.org/opengreyrepository.html
The last search was performed on 2/12/21.
**3) Forwards and backwards searches were performed for all eligible studies and any review articles found on the initial search.**
The forward searching was last updated on 13/2/22.
**4) Conference proceedings abstracts were obtained for**
American Society of Neuroradiology 2010-2021<br>European Congress of Radiology 2014-2016, 2018-2020<br>Barany Society Meeting 2010/2014/2016/2018
These were hand searched on 28/12/21

Supplementary 1: Search strategy
