# Supplementary material for: Delayed post gadolinium MRI descriptors for Meniere’s disease: a systematic review and meta-analysis
Source: Eur Radiol. 2023 May 12;33(10):7113–35. doi: 10.1007/s00330-023-09651-8 (PMC10511628; doi:10.1007/s00330-023-09651-8)
Supplement: Supplementary file 4 — Supplementary file4 (PDF 160 KB) [file 330_2023_9651_MOESM4_ESM.pdf]

Clinical classification	Clinical criteria
**2015 MD Criteria (6)**	
Definite	A. ≥2 spontaneous episodes of vertigo, each lasting 20 minutes to 12 hours<br>B. Audiometrically documented low- to medium-frequency SNHL in affected ear on ≥1 occasion before, during or after 1 of the episodes of vertigo<br>C. Fluctuating aural symptoms (hearing, tinnitus or fullness) in affected ear<br>D. Not better accounted for by another vestibular diagnosis
Probable	A. ≥2 episodes of vertigo or dizziness, each lasting 20 minutes to 24 hours<br>B. Fluctuating aural symptoms (hearing, tinnitus or fullness) in affected ear<br>C. Not better accounted for by another vestibular diagnosis
**1995 MD Criteria (4)**	
Certain Meniere's disease	Definite Meniere's disease, plus histopathologic confirmation
Definite Meniere's disease	A. Two or more definitive spontaneous episodes of vertigo 20 minutes or longer<br>B. Audiometrically documented hearing loss on at least one occasion<br>C. Tinnitus or aural fullness in the treated ear<br>D. Other causes excluded
Probable Meniere's disease	A. One definitive episode of vertigo<br>B. Audiometrically documented hearing loss on at least one occasion<br>C. Tinnitus or aural fullness in the treated ear<br>D. Other causes excluded
Possible Meniere's disease	A. Episodic vertigo of the Meniere type without documented hearing loss, or<br>B. Sensorineural hearing loss, fluctuating or fixed, with dysequilibrium but without definitive episodes<br>C. Other causes excluded

**Monosymptomatic cochlear hydrops (cMD)**	
1972 Criteria (2)	Characterized solely by a fluctuating and progressive sensorineural deafness with all auditory test results typical of MD. Many patients notice a fullness in the ear coincident with the sudden drop in hearing. Some subsequently develop definitive dizzy spells, and the qualifying ‘‘cochlear’’ is discarded
Kimura et al (93)	Fluctuating hearing loss with single episode of vertigo, unsteadiness or no vestibular symptoms
2020 Japanese Clinical Practice Guideline of Meniere’s Disease (7)	Recurrent cochlear symptoms (eg hearing loss, tinnitus, aural fullness) without vertigo attacks with audiometrically demonstrated SNHL (usually low frequency or pan-frequency)
Gurkov (8)	Acute onset low tone acute low tone sudden onset SNHL
Summary criteria applied to meta-analysis for cochlear hydrops	Fluctuating hearing loss or acute low tone sudden onset SNHL with or without aural fullness/tinnitus but no MD type vertigo (eg only single attack of vertigo alone, or with concurrent unsteadiness).
**Monosymptomatic vestibular hydrops (vMD)**	
1972 Criteria (2)	Characterized solely by definitive spells of vertigo. This is more difficult to diagnose as there are no objective findings between spells. The diagnosis may be accepted upon exclusion of other diseases. Some patients subsequently develop deafness, and the qualifying ‘‘vestibular’’ is dropped
Kimura et al (93)	Recurrent episodic vertigo with or without fixed hearing loss
2020 Japanese Clinical Practice	Recurrent attacks of vertigo typical for MD and signs of peripheral dysfunction without accompanying fluctuating cochlear symptoms

Guideline of Meniere's Disease (7)	
Summary criteria for vestibular hydrops applied to meta-analysis	MD type vertigo/episodic vertigo with or without aural fullness/tinnitus and with or without fixed SNHL (but not fluctuating or low frequency).

MD = Meniere's disease; SNHL = sensorineural hearing loss

Supplementary 2: Clinical criteria used to define subgroups in the meta-analysis
