# Supplementary material for: Delayed post gadolinium MRI descriptors for Meniere’s disease: a systematic review and meta-analysis
Source: Eur Radiol. 2023 May 12;33(10):7113–35. doi: 10.1007/s00330-023-09651-8 (PMC10511628; doi:10.1007/s00330-023-09651-8)
Supplement: Supplementary file 5 — Supplementary file5 (PDF 200 KB) [file 330_2023_9651_MOESM5_ESM.pdf]

	Nakashima (96)	Barath(22)	Saccule to utricle ratio index (SURI) (19)	Bernaerts (24)	Kahn (46)	Increased peri-lymphatic enhancement (PLE) (24,46,79)
Cochlea						
Plane and location	**Axial**: Mid-modiolar level	**Axial:** Mid-modiolar level	NA	**Axial:** Mid-modiolar level	**Axial:** Mid-modiolar level	**Axial:** Basal turn and typically inferior segment
Imaging feature/grading	*Grade 1*: cochlear duct area < scala vestibuli area *Grade 2*: cochlear duct > scala vestibuli area*	*Grade 1*: cochlear duct spares part of scala vestibuli *Grade 2:* cochlear duct replaces scala vestibuli*	NA	*Grade 1:* Nodular cochlear duct enlargement "Xmas tree balls" *Grade 2:* Linear cochlear duct enlargement "Xmas garlands" *	Cochlear duct > scala vestibuli area	Asymmetrically increased (in unilateral Meniere's disease) or subjectively increased cochlear PLE
Vestibule						

Plane and location	**Axial:** Inferior aspect of lateral semi-circular canal	**Axial:** At widest part of the vestibule (inferior)	**Sagittal double oblique** (along the longest axial and coronal vestibular dimension) or axial (inferior vestibule)	**Axial**: At widest part of the vestibule (inferior)	Variable	NA
Imaging feature/grading	*Grade 1:* % area of endolymph relative to total fluid area<br>33-50 %<br>*Grade 2:* % area of endolymph relative to total fluid area<br>>50%	*Grade 1:* >50% area of endolymph relative to total fluid area<br>*Grade 2:* enhancing perilymphatic space of the vestibule not visible	Saccule is similar size or larger than the utricle on two references slices.	*Grade 1:* SURI<br>*Grade 2:* utricle and saccule are confluent<br>Grade 3: enhancing perilymphatic space of the vestibule not visible	**Saccule:**<br>*Grade 1:* SURI<br>Grade 2: touching oval window<br>**Utricle:**<br>*Grade 1:* herniation to lateral SCC<br>*Grade 2:* enhancing perilymphatic space of the vestibule not visible<br>**Ampulla:** No surrounding perilymphatic space	NA

<u>Supplementary 3: Description of MRI grading scales incorporating the MRI descriptors</u>
