# Supplementary material for: Delayed post gadolinium MRI descriptors for Meniere’s disease: a systematic review and meta-analysis
Source: Eur Radiol. 2023 May 12;33(10):7113–35. doi: 10.1007/s00330-023-09651-8 (PMC10511628; doi:10.1007/s00330-023-09651-8)
Supplement: Supplementary file 6 — Supplementary file6 (PDF 292 KB) [file 330_2023_9651_MOESM6_ESM.pdf]

Principle reasons for exclusion	
A No data from appropriate control group	n=84
B No data from appropriately defined MD ear group	n=26
C Semiquantitative/qualitative descriptors could not be derived from quantitative data	n=12
D MD ears were selected on the basis of MRI descriptor	n=13
E Conference abstract without enough data	n=5
F No delayed post-gadolinium MRI	n =4
G Only one patient in MD or control group	n=4
H Duplicate data set	n =2
Individual studies: Reasons for exclusion	

**Alonso JE, Ishiyama GP, Fujiwara RJT et al. Cochlear Meniere's: A distinct clinical entity with isolated cochlear hydrops on high-Resolution MRI? Frontiers in Surgery 2021; 8. https://doi.org/10.3389/fsurg.2021.680260**	D
**Attyé A, Dumas G, Troprès I et al. Recurrent peripheral vestibulopathy: Is MRI useful for the diagnosis of endolymphatic hydrops in clinical practice? European Radiology 2015; 25(10): 3043–3049. https://doi.org/10.1007/s00330-015-3712-5**	A
**Bächinger D, Brühlmann C, Honegger, T et al. Endotype-phenotype patterns in Meniere's disease based on gadolinium-enhanced MRI of the vestibular aqueduct. Frontiers in Neurology 2019; 10. https://doi.org/10.3389/fneur.2019.00303**	A
**Bachinger D, Eckhard AH, Roosli C, Veraguth D, Huber A, Dalbert A. Endolymphatic hydrops mimicking obstructive Eustachian tube dysfunction: preliminary experience and literature review. European Archives of Oto-Rhino-Laryngology 2021; 278(2), 561–565. https://doi.org/http://dx.doi.org/10.1007/s00405-020-06139-9**	D
**Bier G, Bongers MN, Schabel C, Heindel W, Ernemann U, Hempel JM. In vivo assessment of an endolymphatic hydrops gradient along the cochlea in patients with Menière's disease by magnetic resonance Imaging — A pilot study. Otol Neurotol 2018; 39(10), e1091–e1099. https://doi.org/10.1097/MAO.0000000000002016**	A
**Bykowski J, Harris JP, Miller M, Du J , Mafee MF. Intratympanic contrast in the evaluation of Menière disease: Understanding the limits. American Journal of Neuroradiology 2015; 36(7), 1326–1332. https://doi.org/10.3174/ajnr.A4277**	A

**Chen W, Chen Y, Geng Y et al. The value of 3D-real IR MRI with intravenous gadolinium injection in the diagnosis of suspected Meniere's disease in children. Acta Oto-Laryngologica 2021; 141(2), 117–121. https://doi.org/10.1080/00016489.2020.1843071**	A
**Chen W, Geng Y, Luo S, Lin N, Sha Y. The correlation of clinical Features and endolymphatic hydrops visualized by 3D-Real IR MRI in children with sudden sensorineural hearing Loss. Ear, Nose and Throat Journal 2021 https://doi.org/10.1177/01455613211009432**	B
**Chen W, Wu X, Geng Y, Lin N, Sha Y. The clinical features and image characteristics of Meniere's disease patients with endolymphatic hydrops confirmed by enhanced magnetic resonance imaging. Brazilian Journal of Otorhinolaryngology 2021 https://doi.org/10.1016/j.bjorl.2021.07.009**	A
**Chen X, Zhang XD, Gu X, Fang ZM, Zhang R. Endolymphatic space imaging in idiopathic sudden sensorineural hearing loss with vertigo. Laryngoscope 2012; 122(10), 2265–2268. https://doi.org/10.1002/lary.23452**	A
**Cho YS, Ahn JM, Choi JE et al. Usefulness of intravenous gadolinium inner ear MR imaging in diagnosis of Ménière's disease. Scientific Reports 2018; 8(1). https://doi.org/10.1038/s41598-018-35709-5**	C
**Cho YS, Kim JS, Kim MB et al. Validation of inner ear MRI in patients with Ménière's disease by comparing endolymphatic hydrops from histopathologic specimens. Scientific Reports 2021; 11(1). https://doi.org/10.1038/s41598-021-97213-7**	A
**Claes G, van den Hauwe L, Wuyts F, van de Heyning P. Does intratympanic gadolinium injection predict efficacy of gentamicin partial chemolabyrinthectomy in Menière's disease patients? Eur Arch Oto-Rhino-Laryngol 2012; 269 (2): 413–418. https://doi.org/10.1007/s00405-011-1644-5**	A

Conte G, Caschera L, Tuscano B et al. Three-Tesla magnetic resonance imaging of the vestibular endolymphatic space: A systematic qualitative description in healthy ears. European Journal of Radiology 2018; 109: 77–82. https://doi.org/10.1016/j.ejrad.2018.10.023	B
de Pont L, van Steekelenburg JM, Verhagen TO. Hydropic Ear Disease: Correlation between audiovestibular symptoms, endolymphatic hydrops and blood-labyrinth barrier impairment. Frontiers in Surgery 2021; 8, 758947. https://doi.org/10.3389/fsurg.2021.758947	D
Dubrulle F, Chaton V, Risoud M, Farah H, Charley Q, Vincent C. The round window sign: a sensitive sign to detect perilymphatic fistulae on delayed postcontrast 3D-FLAIR sequence. Eur Radiol 2020; 30(11): 6303-6310. https://doi.org/10.1007/s00330-020-06924-4	B
Eliezer M, Attyé A, Guichard, JP et al. Vestibular atelectasis: Myth or reality? Laryngoscope 2019; 129(7): 1689–1695. https://doi.org/10.1002/lary.27793	D
Eliezer M, Maquet C, Horion J et al. Detection of intralabyrinthine abnormalities using post-contrast delayed 3D-FLAIR MRI sequences in patients with acute vestibular syndrome. European Radiology 2019; 29(6): 2760–2769. https://doi.org/10.1007/s00330-018-5825-0	B
Eliezer M, Hautefort C, van Nechel C et al. Electrophysiological and inner ear MRI findings in patients with bilateral vestibulopathy. European Archives of Oto-Rhino-Laryngology 2020; 277(5), 1305–1314. https://doi.org/10.1007/s00405-020-05829-8	B
Fiorino F, Pizzini FB, Beltramello A, Barbieri F. MRI performed after intratympanic gadolinium administration in patients with Ménière's disease: Correlation with symptoms and signs. European Archives of Oto-Rhino-Laryngology 2011; 268(2), 181–187. https://doi.org/10.1007/s00405-010-1353-5	A

**Fiorino F, Pizzini FB, Beltramello A, Barbieri F. Progression of endolymphatic hydrops in Ménière's disease as evaluated by Magnetic Resonance Imaging.**	A
**Otol Neurotol 2011; 32: 1152-1157.**	
**Fiorino F, Pizzini B, Barbieri F, Beltramello A. Magnetic resonance imaging fails to show evidence of reduced endolymphatic hydrops in gentamicin treatment of Ménière's disease. Otol Neurotol 2012; 33: 629-633.**	A
**Fiorino F, Pizzini FB, Barbieri F, Beltramello A. Variability in the perilymphatic diffusion of gadolinium does not predict the outcome of intratympanic gentamicin in patients with Ménière's disease. Laryngoscope 2012; 122(4): 907–911. https://doi.org/10.1002/lary.23211**	
**Fiorino F, Mattellini B, Vento M, Mazzocchin L, Bianconi L, Pizzini FB. Does the intravenous administration of frusemide reduce endolymphatic hydrops? Journal of Laryngology and Otology 2016; 130(3): 242–247. https://doi.org/10.1017/S0022215115003527**	A
**Fukuoka H, Tsukada K, Miyagawa M et al. Semi-quantitative evaluation of endolymphatic hydrops by bilateral intratympanic gadolinium-based contrast agent (GBCA) administration with MRI for Meniere's disease. Acta Oto-Laryngologica 2010; 130(1): 10–16. https://doi.org/10.3109/00016480902858881**	C
**Fukuoka H, Takumi Y, Tsukada K et al. Comparison of the diagnostic value of 3 T MRI after intratympanic injection of GBCA, electrocochleography, and the glycerol test in patients with Meniere's disease. Acta Oto-Laryngologica 2010; 132(2): 141–145. https://doi.org/10.3109/00016489.2011.635383**	C

**Fukushima M, Ito R, Miyaguchi S et al. Preceding profound deafness and co-factors promote development of endolymphatic hydrops in preliminary patients with delayed endolymphatic hydrops. Acta Oto-Laryngologica 2016; 136(12): 1304–1308. https://doi.org/10.1080/00016489.2016.1203993**	B
**Fukushima M, Oya R, Akazawa H, Tsuruta Y, Inohara H. Gadolinium-enhanced inner ear magnetic resonance imaging for evaluation of delayed endolymphatic hydrops, including a bilateral case. Acta Oto-Laryngologica 2016; 136(5): 451–455. https://doi.org/10.3109/00016489.2015.1129554**	A
**Fukushima M, Kitahara T, Oya R, Akahani S, Inohara H, Naganawa S ,Takeda N. Longitudinal up-regulation of endolymphatic hydrops in patients with Meniere's disease during medical treatment. Laryngoscope Investigative Otolaryngology 2017; 2(6): 344–350. https://doi.org/10.1002/lio2.115**	C
**Fukushima M, Yokoi K, Iga J, Akahani S, Inohara H, Takeda N. Contralateral type of delayed endolymphatic hydrops may consist of two phenotypes based on a magnetic resonance imaging preliminary study. Acta Oto-Laryngologica 2017; 137(11): 1153–1157. https://doi.org/10.1080/00016489.2017.1347825**	B
**Fukushima M, Akahani S, Inohara H, Takeda N. Stability of endolymphatic hydrops in Meniere disease shown by 3-Tesla magnetic resonance imaging during and after vertigo attacks. JAMA Otolaryngology-Head and Neck Surgery 2019; 145:582-584. doi: 10.1001/jamaoto.2019.0435**	G
**Fukushima M, Oya R, Nozaki K, Eguchi H, Akahani S, Inohara H, Takeda N. Vertical head impulse and caloric are complementary but react opposite to Meniere's disease hydrops. Laryngoscope 2019; 129(7): 1660–1666. https://doi.org/10.1002/lary.27580**	C

**Fujita H, Kitahara T, Koizumi T, Ito T, Inui H, Kakudo M. Investigation of endolymphatic hydrops positivity rates in patients with recurrent audiovestibular symptoms using inner ear magnetic resonance imaging. Auris Nasus Larynx 2021 https://doi.org/10.1016/j.anl.2021.05.009**	A
**Grobman AB, Bhatia R, Angeli SI. Four-hour post-gadolinium MRI for detection of endolymphatic hydrops. Otolaryngology - Head and Neck Surgery (United States). Conference: Annual Meeting of the American Academy of Otolaryngology-Head and Neck Surgery Foundation 2015; 153:99.**	E
**Gu X, Fang ZM, Liu Y, Lin SL, Han B, Zhang R, ChenVX. Diagnostic value of three-dimensional magnetic resonance imaging of inner ear after intratympanic gadolinium injection, and clinical application of magnetic resonance imaging scoring system in patients with delayed endolymphatic hydrops. Journal of Laryngology and Otology 2014; 128(1): 53–59. https://doi.org/10.1017/S0022215113003289**	A
**Gu X, Fang ZM, Liu Y, Huang ZW, Zhang R, Chen X. Diagnostic advantages of intratympanically gadolinium contrast-enhanced magnetic resonance imaging in patients with bilateral Meniere's disease. American Journal of Otolaryngology - Head and Neck Medicine and Surgery 2015; 36(1): 67–73. https://doi.org/10.1016/j.amjoto.2014.10.003**	A
**Guo P, Sun W, Shi S, Zhang F, Wang J, Wang W. Quantitative evaluation of endolymphatic hydrops with MRI through intravenous gadolinium administration and VEMP in unilateral definite Meniere's disease. European Archives of Oto-Rhino-Laryngology 2019; 276(4): 993–1000. https://doi.org/10.1007/s00405-018-05267-7**	A

**Gürkov R, Flatz W, Louza J, Strupp M, Krause E. In vivo visualization of endolyphatic hydrops in patients with Meniere's disease: Correlation with audiovestibular function. European Archives of Oto-Rhino-Laryngology 2011; 268(12): 1743–1748. https://doi.org/10.1007/s00405-011-1573-3**	A
**Gurkov R, Louza J, Strupp M, Flatz W, Krause E. Visualization of endolyphatic hydrops in patients with Meniere's disease by locally enhanced inner ear MRI: Correlation with audiovestibular function. Otolaryngology - Head and Neck Surgery. Conference: Annual Meeting of the American Academy of Otolaryngology-Head and Neck Surgery Foundation 2011. 145(suppl. 2):104.**	A
**Gürkov R, Flatz W, Louza J, Strupp M, Ertl-Wagner B, Krause E. In vivo visualized endolymphatic hydrops and inner ear functions in patients with electrocochleographically confirmed Ménière's disease. Otol Neurotol 2012; 33: 1040-45.**	A
**Gürkov R, Flatz W, Keeser D, Strupp M, Ertl-Wagner B, Krause E. Effect of standard-dose betahistine on endolymphatic hydrops: An MRI pilot study. European Archives of Oto-Rhino-Laryngology 2013;270(4), 1231–1235. https://doi.org/10.1007/s00405-012-2087-3**	A
**Gürkov R , Flatz W, Ertl-Wagner B, Krause E. Endolymphatic hydrops in the horizontal semicircular canal: A morphologic correlate for canal paresis in Ménière's disease. Laryngoscope 2013; 123(2), 503–506. https://doi.org/10.1002/lary.23395**	A
**Gürkov R, Kantner C, Strupp M, Flatz W, Krause E, Ertl-Wagner B. Endolymphatic hydrops in patients with vestibular migraine and auditory symptoms. European Archives of Oto-Rhino-Laryngology 2014; 271(10), 2661–2667. https://doi.org/10.1007/s00405-013-2751-2**	B
**Gürkov R, Berman A , Dietrich O et al. MR volumetric assessment of endolymphatic hydrops. European Radiology 2015; 25(2), 585–595. https://doi.org/10.1007/s00330-014-3414-4**	A

**Gürkov R, Todt I, Jadeed R, Sudhoff H, Gehl HB. Laterality of audiovestibular symptoms predicts laterality of endolymphatic hydrops in hydropic ear disease (Menière). Otol Neurotol 2020; 41(9), e1140–e1144. https://doi.org/10.1097/MAO.0000000000002775**	A
**Gurkov R, Lutsenko V, Situkho M, Babkina T, Valchyshyn S. Clinical high-resolution imaging and grading of endolymphatic hydrops in hydropic ear disease at 1.5 T using the two-slice grading for vestibular endolymphatic hydrops in less than 10 min. European Archives of Oto-Rhino-Laryngology 2021 https://doi.org/http://dx.doi.org/10.1007/s00405-021-06731-7**	A
**He J, Peng A, Hu J et al. Dynamics in endolymphatic hydrops and symptoms in Meniere's disease after endolymphatic duct blockage, preliminary results. Frontiers in Neurology 2021; 11. https://doi.org/10.3389/fneur.2020.622760**	A
**He B, Zhang F, Zheng H et al. The correlation of a 2D volume-referencing endolymphatic-hydrops grading system with extra-tympanic electrocochleography in patients with definite Ménière's disease. Frontiers in Neurology 2021; 11. https://doi.org/10.3389/fneur.2020.595038**	A
**Heider C, Plontke S, Gotze G, Rahne T, Kosling S. Clinical application of MR imaging with contrast-based 3D IR-sequence at Meniere Disease. Laryngol-Rhino-Otologie Conference HNO 2018; 97 (suppl 2):S172-S173**	A
**Higashi-Shingai K, Imai T, Okumura T et al. Change in endolymphatic hydrops 2 years after endolymphatic sac surgery evaluated by MRI. Auris Nasus Larynx 2019; 46(3): 335–345. https://doi.org/10.1016/j.anl.2018.10.011**	A

**Homann G, Vieth V, Weiss D, Nikolaou K, Heindel W, Notohamiprodjo M, Böckenfeld Y. Semi-quantitative vs. volumetric determination of endolymphatic space in Menière's disease using endolymphatic hydrops 3T-HR-MRI after intravenous gadolinium injection. PLoS ONE 2015; 10(3).**	B
**https://doi.org/10.1371/journal.pone.0120357**	
**Hornibrook J, Coates M, Goh T, Bird P. MRI imaging of the inner ear for Meniere's disease. N Z Med J 2010; 123(1321):59–63.**	A
**PMID: 20927159**	
**Hornibrook J, Coates M, Goh A, Gourley J, Bird P. Magnetic resonance imaging for Ménière's disease: Correlation with tone burst electrocochleography. Journal of Laryngology and Otology 2012; 126(2): 136–141. https://doi.org/10.1017/S0022215111003112**	A
**Hornibrook J, Flook E, Greig S et al. MRI inner ear imaging and tone burst electrocochleography in the diagnosis of Ménière's disease. Otol Neurotol 2015; 36 (6):1109-1114. doi: 10.1097/mao.0000000000000782**	A
**Herrera I, Costa CA, Espanol CC, Bordn JM, Gonzalez CO, Guijarro BS. Detection of endolymphatic hydrops in Meniere disease using 3T MRI after intravenous administration of gadolinium. 47th Annual Meeting of the Spanish Society of Neuroradiology, SENR 2018; 61(4):491.**	E
**Iida T, Teranishi M, Yoshida T et al. Magnetic resonance imaging of the inner ear after both intratympanic and intravenous gadolinium injections. Acta Oto-Laryngologica 2013; 133(5): 434–438. https://doi.org/10.3109/00016489.2012.753640**	H

**Inui H, Sakamoto T, Ito T, Kitahara T. Magnetic resonance imaging of endolymphatic space in patients with sensorineural hearing loss: comparison between fluctuating and idiopathic sudden sensorineural hearing loss. Acta Oto-Laryngologica 2020; 140(5): 345–350. https://doi.org/10.1080/00016489.2020.1720919**	C
**Ito T, Inui H, Miyasaka T et al. Relationship between changes in hearing function and volumes of endolymphatic hydrops after endolymphatic sac drainage. Acta Oto-Laryngologica 2019; 139(9): 739–746. https://doi.org/10.1080/00016489.2019.1630757**	A
**Ito T, Inui H, Miyasaka T et al. Endolymphatic volume in patients with Meniere's disease and healthy controls: Three-dimensional analysis with magnetic resonance imaging. Laryngoscope Investigative Otolaryngology 2019; 4(6): 653–658. https://doi.org/10.1002/lio2.313**	C
**Ito T, Inui H, Miyasaka T et al. Three-dimensional magnetic resonance imaging reveals the relationship between the control of vertigo and decreases in endolymphatic hydrops after endolymphatic sac drainage with steroids for Meniere's disease. Frontiers in Neurology 2019; 10. https://doi.org/10.3389/fneur.2019.00046**	A
**Ito T, Inoue T, Inui H et al. Novel magnetic resonance imaging-based method for accurate diagnosis of Meniere's disease. Frontiers in Surgery 2021; 8. https://doi.org/10.3389/fsurg.2021.671624**	C
**Iwasa Y-I, Tsukada K, Kobayashi M et al. Bilateral delayed endolymphatic hydrops evaluated by bilateral intratympanic injection of gadodiamide with 3T-MRI. PLoS ONE 2018; 13(12). https://doi.org/10.1371/journal.pone.0206891**	B
**Kato K, Yoshida T, Teranishi M et al. Peak width in multifrequency tympanometry and endolymphatic hydrops revealed by magnetic resonance imaging. Otol Neurotol 2012; 33(6): 912-5.doi: 10.1097/MAO.0b013e31825d9a72**	A

**Kato M, Sugiura M, Shimono M et al. Endolymphatic hydrops revealed by magnetic resonance imaging in patients with atypical Meniere's disease. Acta Oto-Laryngologica 2013 133(2): 123–129. https://doi.org/10.3109/00016489.2012.726374**	A
**Kato M, Teranishi M, Katayama N, Sone M, Naganawa S, Nakashima T. Association between endolymphatic hydrops as revealed by magnetic resonance imaging and caloric response. Otology Neurotol 32(9): 1480-5. doi:10.1097/MAO.0b013e318235568d**	A
**Kim TY, Park DW, Lee YJ, Lee JY, Lee SH, Chung JH, Lee S. Comparison of inner ear contrast enhancement among patients with unilateral inner ear symptoms in MR images obtained 10 minutes and 4 hours after gadolinium injection. AJNR 2015; 36(12): 2367–2372. https://doi.org/10.3174/ajnr.A4439**	B
**Kirsch V, Nejatbakhshesfahani F, Ahmadi SA, Dieterich M, Ertl-Wagner B. A probabilistic atlas of the human inner ear's bony labyrinth enables reliable atlas-based segmentation of the total fluid space. Journal of Neurology 2019; 266: 52–61. https://doi.org/10.1007/s00415-019-09488-6**	D
**Kitano K, Kitahara T, Ito T, Shiozaki T, Wada Y, Yamanaka T. Results in caloric test, video head impulse test and inner ear MRI in patients with Ménière's disease. Auris Nasus Larynx 2020; 47(1): 71–78. https://doi.org/10.1016/j.anl.2019.06.002**	A
**Lee J, Kim ES, Lee Y et al. Quantitative analysis of cochlear signal intensity on three-dimensional and contrast-enhanced fluid-attenuated inversion recovery images in patients with Meniere's disease: Correlation with the pure tone audiometry test. Journal of Neuroradiology 2019; 46(5): 307–311. https://doi.org/10.1016/j.neurad.2019.03.010**	F

**Li Y, Sha Y, Wang F et al. Comprehensive comparison of MR image quality between intratympanic and intravenous gadolinium injection using 3D real IR sequences. Acta Oto-Laryngologica 2019;139(8): 659–664. https://doi.org/10.1080/00016489.2019.1600719**	A
**Liu F, Huang W, Chen Q, Meng X, Wang Z, He Y. Non-invasive evaluation of the effect of endolymphatic sac decompression in Ménière's disease using magnetic resonance imaging. Acta Oto-Laryngologica 2014; 134(7): 666–671. https://doi.org/10.3109/00016489.2014.885118**	A
**Liu Y, Zhang F, He B, He J, Zhang Q, Yang J, Duan M. Vestibular endolymphatic hydrops visualized by magnetic resonance imaging and its correlation with vestibular functional test in patients with unilateral Meniere's disease. Frontiers in Surgery 2021; 8. https://doi.org/10.3389/fsurg.2021.673811**	A
**Louza JPR, Flatz W, Krause E , Gurkov R. Short-term audiologic effect of intratympanic gadolinium contrast agent application in patients with Meniere's disease. American Journal of Otolaryngology - Head and Neck Medicine and Surgery 2012; 33(5):533-53.**	A
**Louza J, Krause E, Gurkov R. Audiologic evaluation of Meniere's disease patients one day and one week after intratympanic application of gadolinium contrast agent: Our experience in sixty-five patients. Clinical Otolaryngology 2013; 38(3): 262-266.**	C
**Louza J, Krause E, Gurkov R. Hearing function after intratympanic application of gadolinium-based contrast agent: A long-term evaluation. Laryngoscope 2015; 125(10):2366-2370**	A
**Luzeiro I, MacHado R, Silva B, Pereira D. The role of endolymphatic hydrops in differentiating vestibular migraine from Meniere's disease. Headache. Conference: 63rd American Headache Society Annual Scientific Meeting, AHS 2021; 61:168.**	E

**Maxwell AK, Ishiyama G, Karnezis S, Ishiyama A. Isolated saccular hydrops on high-resolution MRI is associated with full spectrum Menière's Disease. Otology & Neurotology 2021; 42(6): 876–882. https://doi.org/10.1097/MAO.0000000000003051**	D
**Min XH, Gu H, Zhang Y, Li K, Pan ZY, Jiang T. Clinical value of abnormal MRI findings in patients with unilateral sudden sensorineural hearing loss. Diagn Interv Radiol 2020; 26(5): 429-436. doi: 10.5152/dir.2020.19229**	B
**Morioka M, Sugimoto S, Yoshida T et al. Dilatation of the endolymphatic space in the ampulla of the posterior semicircular canal: A new clinical finding detected on magnetic resonance imaging. Otol Neurotol 2021; 42(6) e643–e647. https://doi.org/10.1097/MAO.0000000000003073**	D
**Naganawa S, Koshikawa T, Fukatsu H, Ishigaki T, Nakashima T, Ichinose N. Contrast-enhanced MR imaging of the endolymphatic sac in patients with sudden hearing loss. European Radiology 2002; 12(5): 1121–1126. https://doi.org/10.1007/s00330-001-1216-y**	F
**Naganawa S, Satake H, Kawamura M, Fukatsu H, Sone M, Nakashima T. Separate visualization of endolymphatic space, perilymphatic space and bone by a single pulse sequence; 3D-inversion recovery imaging utilizing real reconstruction after intratympanic Gd-DTPA administration at 3 Tesla. European Radiology 2008; 18(5): 920–924. https://doi.org/10.1007/s00330-008-0854-8**	A
**Naganawa S, Satake H, Iwano S, Fukatsu H, Sone M, Nakashima T. Imaging endolymphatic hydrops at 3 Tesla using 3D-FLAIR with intratympanic Gd-DTPA administration. Magn Reson Med Sci 2008; 7(2): 85-91.**	G

**Naganawa S, Sugiura M, Kawamura M, Fukatsu H, Sone M, Nakashima T. Imaging of endolymphatic and perilymphatic fluid at 3T after intratympanic administration of gadolinium-diethylene-triamine pentaacetic acid. American Journal of Neuroradiology 2008; 29(4): 724–726. https://doi.org/10.3174/ajnr.A0894**	G
**Naganawa S, Ishihara S, Iwano S, Sone M, Nakashima T. Three-dimensional (3D) visualization of endolymphatic hydrops after intratympanic injection of Gd-DTPA: Optimization of a 3D-real inversion-recovery turbo spin-echo (TSE) sequence and application of a 32-channel head coil at 3T. Journal of Magnetic Resonance Imaging 2010; 31(1), 210–214. https://doi.org/10.1002/jmri.22012**	B
**Naganawa S, Ishihara S, Iwano S, Kawai H., Sone M, Nakashima T. Estimation of gadolinium-induced T1-shortening with measurement of simple signal intensity ratio between the cochlea and brain parenchyma on 3D-FLAIR: Correlation with T1 measurement by TI scout sequence. Magn Reson Med Sci 2010; 9(1): 17-22. doi:10.2463/MRMS.9.17.**	B
**Naganawa S, Yamazaki M, Kawai H, Bokura K, Sone M, Nakashima T. Visualization of endolymphatic hydrops in Meniere's disease with single-dose intravenous gadolinium-based contrast media using heavily T2-weighted 3D-FLAIR. Magn Reson Med Sci 2010; 9(4): 237-42. doi:10.2463/MRMS.9.237.**	A
**Naganawa S, Kawai H, Sone M, Nakashima T. Increased sensitivity to low concentration gadolinium contrast by optimized heavily T2-weighted 3D-FLAIR to visualize endolymphatic space. Magn Reson Med Sci 2010; 9(2): 73-80. doi: 10.2463/mrms.9.73.**	B
**Naganawa S, Sone M, Yamazaki M, Kawai H., Nakashima T. Visualization of endolymphatic hydrops after intratympanic injection of Gd-DTPA: Comparison of 2D and 3D Real inversion recovery imaging. Magn Reson Med Sci 2011; 10(2):415-20. doi: 10.1097/MLG.0b013e31802c300c.**	A

**Naganawa S, Yamazaki M, Kawai H, Bokura K, Sone M, Nakashima T. Imaging of Meniere ’s disease after intravenous administration of single-dose gadodiamide: Utility of subtraction images with different inversion time. Magn Reson Med Sci 2012;11 (3):213-9. doi: 10.2463/mrms.11.213.**	B
**Naganawa S, Yamazaki M, Kawai H, Bokura K, Sone M, Nakashima T. Imaging of Meniere’s disease by subtraction of MR Cisternography from positive perilymph Image. Magn Reson Med Sci 2012; 11(4): 303-309. https://doi.org/10.2463/mrms.11.303**	A
**Naganawa S, Suzuki K, Nakamichi R et al. Semi-quantification of endolymphatic size on MR imaging after intravenous injection of single dose gadodiamide: Comparison between two types of processing strategies. Magn Reson Med Sci 2013; 12(4):261-269. doi: 10.2463/mrms.2013-0019.**	C
**Naganawa S, Yamazaki M, Kawai H, Bokura K, Sone M, Nakashima T. Estimation of perilymph enhancement after intratympanic administration of Gd-DTPA by fast T1-mapping with a dual flip angle 3D spoiled gradient echo sequence. Magn Reson Med Sci 2013; 12(3): 223-228. doi:10.2463/mrms.2012-0071**	A
**Naganawa S, Yamazaki M, Kawai H, Bokura K, Sone M., Nakashima T. Imaging of Ménière’s disease after intravenous administration of single-dose gadodiamide: Utility of multiplication of MR cisternography and HYDROPS image. Magn Reson Med Sci 2013; 12(1), 63–68. https://doi.org/10.2463/mrms.2012-0027**	A

**Naganawa S, Yamazaki M, Kawai H, Bokura K, Sone M, Nakashima T. Visualization of endolymphatic hydrops in Méniere's disease after intravenous administration of single-dose gadodiamide at 1.5T. Magn Reson Med Sci 2013; 12(2), 137–139. https://doi.org/10.2463/mrms.2012-0037**	A
**Naganawa S, Yamazaki M, Kawai H, Bokura K, Iida T, Sone M, Nakashima T. MR imaging of Ménière's disease after combined intratympanic and intravenous injection of gadolinium using hydrops 2. Magn Reson Med Sci 2014; 13(2), 133–137. https://doi.org/10.2463/mrms.2013-0061**	A
**Naganawa S, Kawai H, Ikeda M, Sone M, Nakashima T. Imaging of endolymphatic hydrops in 10 minutes: A new strategy to reduce scan time to one third. Magn Reson Med Sci 2015 14(1), 77–83. https://doi.org/10.2463/mrms.2014-0065.**	A
**Naganawa S, Ohashi T, Kanou M, Kuno K, Sone M, Ikeda M. Volume quantification of endolymph after intravenous administration of a single dose of gadolinium contrast agent: Comparison of 18- versus 8-minute imaging protocols. Magn Reson Med Sci 2015; 14(4): 257–262. https://doi.org/10.2463/mrms.2014-0118**	B
**Naganawa S, Kanou M, Ohashi T, Kuno K, Sone M. Simple estimation of the endolymphatic volume ratio after intravenous dministration of a single-dose of gadolinium contrast. Magnetic resonance in medical sciences: MRMS: an official journal of Japan Society of Magnetic Resonance in Medicine 2016; 15:379-385. doi:10.2463/mrms.mp.2015-0175**	A
**Naganawa S, Kawai H, Taoka T, Sone M. Improved HYDROPS: Imaging of Endolymphatic Hydrops after Intravenous Administration of Gadolinium. Magnetic resonance in medical sciences: MRMS : an official journal of Japan Society of Magnetic Resonance in Medicine 2017;16(4):357-361. doi: 10.2463/mrms.tn.2016-0126**	C

**Naganawa S, Ito R, Kawai H, Kawamura M, Taoka T, Yoshida T, Sone M. Cross-sectional area of the superior petrosal sinus is reduced in patients with significant endolymphatic hydrops. Magn Reson Med Sci 2021; https://doi.org/10.2463/mrms.mp.2021-0010**	A
**Naganawa S, Nakamichi R, Ichikawa K, Kawamura M, Kawai H, Yoshida T, Sone M. MR imaging of endolymphatic hydrops: Utility of iHYDROPS-Mi2 combined with deep learning reconstruction denoising. Magn Reson Med Sci 2021: 20(3): 272–279. https://doi.org/10.2463/mrms.mp.2020-0082**	A
**Nakashima T, Naganawa S, Katayama N et al. Clinical significance of endolymphatic imaging after intratympanic gadolinium injection. Acta Oto-Laryngologica 2009; 129(560): 9–14. https://doi.org/10.1080/00016480902729801**	A
**Nakashima T, Naganawa S, Sugiura M et al. Visualization of endolymphatic hydrops in patients with Meniere's disease. Laryngoscope 2007; 117(3): 415–420. https://doi.org/10.1097/MLG.0b013e31802c300c**	A
**Nakashima T, Naganawa S, Teranishi M et al. Endolymphatic hydrops revealed by intravenous gadolinium injection in patients with Ménière's disease. Acta Oto-Laryngologica 2010; 130(3): 338–343. https://doi.org/10.3109/00016480903143986**	A
**Neri G, Tartaro A, Neri L. MRI with intratympanic gadolinium: Comparison between otoneurological and radiological investigation in Menière's disease. Frontiers in Surgery 2021; 8. https://doi.org/10.3389/fsurg.2021.672284**	A

**Nonoyama H, Tanigawa T, Tamaki T, Tanaka H, Yamamuro, O, Ueda H. Evidence for bilateral endolymphatic hydrops in ipsilateral delayed endolymphatic hydrops: Preliminary results from examination of five cases. Acta Oto-Laryngologica 2014; 134(3), 221–226. https://doi.org/10.3109/00016489.2013.850741**	B
**Ohashi T, Naganawa S, Takeuchi A, Katagir T, Kuno K. Quantification of endolymphatic space volume after intravenous administration of a single dose of gadolinium-based contrast agent: 3D-real inversion recovery versus HYDROPS-Mi2. Magn Reson Med Sci2020; 19(2), 119–124. https://doi.org/10.2463/mrms.mp.2019-0013**	A
**Okumura T, Imai T, Takimoto Y et al. Assessment of endolymphatic hydrops and otolith function in patients with Ménière's disease. European Archives of Oto-Rhino-Laryngology 2017; 274(3): 1413–1421. https://doi.org/10.1007/s00405-016-4418-2**	A
**Osman S, Hautefort C, Attyé A , Vaussy, A , Houdart E, Eliezer M. Increased signal intensity with delayed post contrast 3D-FLAIR MRI sequence using constant flip angle and long repetition time for inner ear evaluation: Increased sensitivity to low concentration of gadolinium for inner ear MRI. Diagnostic and Interventional Imaging 2021. https://doi.org/10.1016/j.diii.2021.10.003**	B
**Oya R, Imai T, Sato T et al. A high jugular bulb and poor development of perivestibular aqueductal air cells are not the cause of endolymphatic hydrops in patients with Meniere's disease. Auris Nasus Larynx 2018; 45(4): 693-701.**	D
**Pauna HF, Gasperin A, Coelho LO. Correlation between enhanced MRI and audio-vestibular tests in Meniere's disease. Otolaryngology - Head and Neck Surgery. Conference: AAO-HNSF 2021 Annual Meeting and OTO Experience 2021; 165:250.**	E

**Paškoniene A, Baltagalviene R, Lengvenis G et al. The Importance of the Temporal Bone 3T MR Imaging in the Diagnosis of Menière's Disease. Otology and Neurotology 2020; 41(2): 235–241. https://doi.org/10.1097/MAO.0000000000002471**	A
**Peng A, Hu J, Wang, Q et al. Modulation of hearing function following the downgrading or upgrading of endolymphatic hydrops in Meniere's disease patients with endolymphatic duct blockage. PLoS ONE 2020; 15(10). https://doi.org/10.1371/journal.pone.0240315**	A
**Samoy K, Casselman J, Haspeslagh M, Timmermans M, Kuhweide R, Lerut B, Vinck AS. Gadolinium-enhanced inner ear magnetic resonance imaging for evaluation of endolymphatic hydrops: Correlation with audiometric testing and auditory symptoms. B-ENT. Conference: Annual Meeting of the Royal Belgian Society for Ear, Nose and Throat, Head and Neck Surgery 2017; 13 (suppl 27):25**	E
**Seo YJ, Kim J, Choi JY, Lee WS. Visualization of endolymphatic hydrops and correlation with audio-vestibular functional testing in patients with definite Meniere's disease. Auris Nasus Larynx 2013; 40(2), 167–172. https://doi.org/10.1016/j.anl.2012.07.009**	A
**Sepahdari AR, Ishiyama G, Vorasubin N, Peng KA, Linetsky M, Ishiyama A. Delayed intravenous contrast-enhanced 3D FLAIR MRI in Meniere's disease: Correlation of quantitative measures of endolymphatic hydrops with hearing. Clinical Imaging 2015; 39(1): 26–31. https://doi.org/10.1016/j.clinimag.2014.09.014**	A
**Sepahdari A, Vorasubin N, Ishiyama G., Ishiyama A. Endolymphatic hydrops reversal following acetazolamide therapy: Demonstration with delayed intravenous contrast-enhanced 3D-FLAIR MRI. American Journal of Neuroradiology 2016; 37(1): 151–154. https://doi.org/10.3174/ajnr.A4462**	D

**Shi H, Li Y, Yin S, Zou J. The predominant vestibular uptake of gadolinium through the oval window pathway is compromised by endolymphatic hydrops in Ménière's disease. Otol Neurotol 2014; 35(2): 315-322. doi: 10.1097/MAO.0000000000000196**	G
**Shi S, Zhou F, Wang W. 3D-real IR MRI of Meniere's disease with partial endolymphatic hydrops. Am J Otol 2019; 40 (4): 589-593. https://doi.org/10.1016/j.amjoto.2019.05.015.**	D
**Shi S, Zhou F, Wang W. 3D-real IR MRI of Meniere's disease with partial endolymphatic hydrops. American Journal of Otolaryngology - Head and Neck Medicine and Surgery 2019 40(4); 589–593. https://doi.org/10.1016/j.amjoto.2019.05.015**	A
**Suga K, Kato M, Yoshida T et al. Changes in endolymphatic hydrops in patients with Ménière's disease treated conservatively for more than 1 year. Acta Oto-Laryngologica 2015; 135(9): 866–870. https://doi.org/10.3109/00016489.2015.1015607**	A
**Sugimoto S, Yoshida T, Teranishi M, Okazaki Y, Naganawa S, Sone M. The relationship between endolymphatic hydrops in the vestibule and low-frequency air-bone gaps. Laryngoscope 2018; 128(7): 1658–1662. https://doi.org/10.1002/lary.26898**	D
**Sugimoto S, Yoshida T, Teranishi M, Kobayashi M, Shimono M, Naganawa S, Sone M. Significance of endolymphatic hydrops herniation into the semicircular canals detected on MRI. Otol Neurotol 2018; 39(10), 1229–1234. https://doi.org/10.1097/MAO.0000000000002022**	D
**Sugiura M, Naganawa S, Nakashima T, Misawa H, Nakamura T. Magnetic resonance imaging of endolymphatic sac in acute low-tone sensorineural hearing loss without vertigo. ORL 2003; 65(5):254-260. doi: 10.1159/000075222**	F

**Suzuki H, Teranishi M, Sone M, Yamazaki M, Naganawa S, Nakashima T. Contrast enhancement of the inner ear after intravenous administration of a standard or double dose of gadolinium contrast agents. Acta Oto-Laryngologica 2011; 131(10):1025-1031.**	C
**Suzuki H, Teranishi M, Naganawa S, Nakata S, Sone M., Nakashima T. Contrast-enhanced MRI of the inner ear after intratympanic injection of meglumine gadopentetate or gadodiamide hydrate. Acta Oto-Laryngologica 2011; 131(2): 130–135. https://doi.org/10.3109/00016489.2010.507781**	A
**Tagaya M, Teranishi M, Naganawa S et al. 3 Tesla magnetic resonance imaging obtained 4 hours after intravenous gadolinium injection in patients with sudden deafness. Acta Oto-Laryngologica 2010; 130(6): 665–669. https://doi.org/10.3109/00016480903384176**	B
**Tanigawa T, Tanaka H, Sato T et al. 3D-FLAIR MRI findings in patients with low-tone sudden deafness. Acta Oto-Laryngologica 2010; 130(12): 1324–1328. https://doi.org/10.3109/00016489.2010.496461**	F
**Teranishi M, Naganawa S, Katayama N, Sugiura M, Nakata S, Sone M, Nakashima T. Image evaluation of endolymphatic space in fluctuating hearing loss without vertigo. European Archives of Oto-Rhino-Laryngology 2009; 266(12):1871–1877. https://doi.org/10.1007/s00405-009-0989-5**	A
**Tuñón Gómez M, Lobo Duro DR, Brea Álvarez B, García-Berrocal JR. Diagnosis of endolymphatic hydrops by means of 3 T magnetic resonance imaging after intratympanic administration of gadolinium. Radiología (English Edition) 2017; 59(2): 159–165. https://doi.org/10.1016/j.rxeng.2017.02.001**	A

**Uno A, Imai T, Watanabe Y et al. Changes in endolymphatic hydrops after sac surgery examined by Gd-enhanced MRI. Acta Oto-Laryngologica 2013; 133(9), 924–929. https://doi.org/10.3109/00016489.2013.795290**	A
**Van Steekelenburg J, Van Weijnen A, Vijlbrief O, Blom H, Hammer S. Accuracy of 3DIR MRI in Meniere's disease. Neuroradiology 2018; 60 (Suppl 2): s452 https://doi.org/10.1007/s00234-018-2057-6**	H
**Wang J, Ren T, Sun W, Liang Q, Wang W. Post-contrast 3D-FLAIR in idiopathic sudden sensorineural hearing loss. Eur Arch Otorhinolaryngol 2019; 276(5): 1291-1299. doi: 10.1007/s00405-019-05285-z**	B
**Wang P, Yu D, Wang H et al. Contrast-enhanced MRI combined with the glycerol test reveals the heterogeneous dynamics of endolymphatic hydrops in patients with Menière's disease. Journal of Magnetic Resonance Imaging 2012; 52(4), 1066–1073. https://doi.org/10.1002/jmri.27127**	A
**Wesseler A, Óvári A, Javorkova A, Kwiatkowski A, Meyer JE, Kivelitz DE Diagnostic value of the magnetic resonance imaging with intratympanic gadolinium administration (IT-Gd MRI) versus audio-vestibular tests in Menière's disease: IT-Gd MRI makes the difference. Otol Neurotol 2019; 40(3), E225–E232. https://doi.org/10.1097/MAO.0000000000002082**	A
**Wu Q, Li X, Sha Y, Dai C. Clinical features and management of Meniere's disease patients with drop attacks. European Archives of Oto-Rhino-Laryngology 2019; 276(3), 665–672. https://doi.org/10.1007/s00405-018-5260-5**	A

**Xie J, Zhang W, Zhu J, Hui L, Li S, Zhang B. Comparison of inner ear MRI enhancement in patients with Meniere's disease after intravenous injection of gadobutrol, gadoterate meglumine, or gadodiamide. European Journal of Radiology 2021; 139. https://doi.org/10.1016/J.EJRAD.2021.109682**	D
**Yamamoto M, Teranishi M, Naganawa S et al. Relationship between the degree of endolymphatic hydrops and electrocochleography. Audiology and Neurotology 2010; 15(4): 254–260. https://doi.org/10.1159/000258681**	A
**Yamazaki M, Naganawa S, Kawai H, Nihashi T, Nakashima T. Signal alteration of the cochlear perilymph on 3 different sequences after intratympanic Gd-DTPA administration at 3 Tesla: Comparison of 3D-FLAIR, 3D-T1-weighted Imaging, and 3D-CISS. Magn Reson Med Sci 2010; 9(2):65-71. doi: 10.2463/mrms.9.65.**	A
**Yamazaki M, Naganawa S, Tagaya M et al. Comparison of contrast effect on the cochlear perilymph after intratympanic and intravenous gadolinium injection. American Journal of Neuroradiology 2012; 33(4), 773–778. https://doi.org/10.3174/ajnr.A2821.**	A
**Yamazaki M, Naganawa S, Kawai H, Sone M, Nakashima T. Gadolinium distribution in cochlear perilymph: Differences between intratympanic and intravenous gadolinium injection. Neuroradiology 2012; 54(10), 1161–1169. https://doi.org/10.1007/s00234-012-1078-9**	A
**Yang S, Zhu H, Zhu B et al. Correlations between the degree of endolymphatic hydrops and symptoms and audiological test results in patients with Menière's disease: A re-evaluation. Otol Neurotol 2018; 39(3): 351–356. https://doi.org/10.1097/MAO.0000000000001675**	A

Yoshida T, Teranishi M, Kato M et al. Endolymphatic hydrops in patients with tinnitus as the major symptom. European Archives of Oto-Rhino-Laryngology 2013; 270(12): 3043–3048. https://doi.org/10.1007/s00405-013-2380-9	B
Yoshioka M, Naganawa S, Sone M, Nakata S, Teranishi M, Nakashima T. Individual differences in the permeability of the round window: Evaluating the movement of intratympanic gadolinium into the inner ear. Otol Neurotol 2009; 30: 645-648. doi: 10.1097/MAO.0b013e31819bda66	B
Yu J, Zhou YJ, Xu X da et al Different findings of morphological changes and functional decline in the vestibule and the semicircular canal in ipsilateral delayed endolymphatic hydrops. Clinical Neurophysiology 2020; 131(7), 1487–1494. https://doi.org/10.1016/j.clinph.2020.03.032	B
Zhang Y, Cui Y hua, Hu Y. Changes in endolymphatic hydrops visualized by magnetic resonance imaging after sac surgery. Journal of Huazhong University of Science and Technology - Medical Science 2016; 36(5): 736–740. https://doi.org/10.1007/s11596-016-1654-z	A
Zhang W, Hui L, Zhang B, Ren L, Zhu J, Wang F, Li S. The Correlation Between Endolymphatic Hydrops and Clinical Features of Meniere Disease. Laryngoscope 2021; 131(1): e144–E150. https://doi.org/10.1002/lary.28576	C
Zhang W, Xie J, Hui L, Li S, Zhang B. The Correlation Between Endolymphatic Hydrops and blood-labyrinth barrier Permeability of Meniere Disease. Annals of Otology, Rhinology and Laryngology 2021; 130(6): 578–584. https://doi.org/10.1177/0003489420964823	A

**Zheng Y, Liu A, Wang X long, Hu Y, Zhang Y, Peng L. The role of endolymphatic hydrops in patients with pantonal idiopathic sudden sensorineural hearing loss: A cause or secondary reaction. Current Medical Science 2019; 39(6): 972–977. https://doi.org/10.1007/s11596-019-2130-3**	B
**Zhu HL, Ou YK, Fu J, Zhang Y, Xiong H, Xu YD. A comparison of inner ear imaging features at different time points of sudden sensorineural hearing loss with three-dimensional fluid-attenuated inversion recovery magnetic resonance imaging. Eur Arch Otorhinolaryngol 2015; 272(10): 2659-65. doi: 10.1007/s00405-014-3187-z.**	B
**Zou J, Chen L, Li H, Zhang G, Pyykkö, I, Lu J. High-quality imaging of endolymphatic hydrops acquired in 7 minutes using sensitive hT2W–3D–FLAIR reconstructed with magnitude and zero-filled interpolation. European Archives of Oto-Rhino-Laryngology 2021; https://doi.org/10.1007/s00405-021-06912-4**	A

Supplementary 4: Reasons for exclusions of database and register reports after full text review for eligibility
