# Supplementary material for: Delayed post gadolinium MRI descriptors for Meniere’s disease: a systematic review and meta-analysis
Source: Eur Radiol. 2023 May 12;33(10):7113–35. doi: 10.1007/s00330-023-09651-8 (PMC10511628; doi:10.1007/s00330-023-09651-8)
Supplement: Supplementary file 7 — Supplementary file7 (PDF 158 KB) [file 330_2023_9651_MOESM7_ESM.pdf]

**Patient selection**
Risk of Bias
Could the selection of patients have introduced bias? (Low/High/Unclear risk)
• Was a consecutive or random sample enrolled?
• Was a case control methodology avoided?
• Did the study avoid inappropriate exclusions?
Notes: Inappropriate exclusions to introduce risk of bias include previous intratympanic gentamicin, specific duration of MD, finding of other pathology on MRI and bilateral MD ears
Concerns regarding applicability
• Are there concerns that the included patients and setting do not match the review question? (Low/High/Unclear risk)
Notes: Applicability concerns introduced when all clinically suspected MD ears were not included in the cohort
**Conduct and interpretation of index test**
Risk of Bias
Could the conduct or interpretation of the index test introduce bias? (Low/High/Unclear risk)
Notes: When only MD ears were included, it was not possible for the observers to be blinded to the diagnosis. The threshold was generally pre-specified unless quantitative area data was used to derive the MRI descriptor.
Concerns regarding applicability
Are there concerns that the index test or its conduct or interpretation differs from the review question? (Low/High/Unclear risk)
Notes: All selected studies included delayed post gadolinium MRI studies either by IV or IT route.
**Reference standard conduct and interpretation**
Risk of bias
Could the reference standard or its conduct or interpretation have introduced bias? (Low/High/unclear risk)
• Are the reference standards likely to correctly classify the target condition?
• Was the reference standard's result interpreted without knowledge of the index test?
Notes: If the reference standard was not the definite 2015 clinical criteria, then this was considered to potentially misclassify the target condition.
Concerns regarding applicability
Are there concerns that the target condition as defined by the reference standard does not match the review question? (Low/High/Unclear risk)

**Flow and timing**
Risk of bias
Could the patient flow have introduced bias? (Low/High/Unclear risk)
• Was there an appropriate interval between index test and reference standard?
• Did all patients receive same reference standard?
• Were all patients included in analysis?
Notes: An appropriate interval index test and reference standard was only considered low risk if a prospective study. If a range of different clinical criteria were included in the study, then the patients were not deemed to receive the same reference standard. Post hoc exclusions such as technically inadequate MRI was considered high risk of bias.

Supplementary 5: QUADAS 2 questions and notes
