# Supplementary material for: Delayed post gadolinium MRI descriptors for Meniere’s disease: a systematic review and meta-analysis
Source: Eur Radiol. 2023 May 12;33(10):7113–35. doi: 10.1007/s00330-023-09651-8 (PMC10511628; doi:10.1007/s00330-023-09651-8)
Supplement: Supplementary file 8 — Supplementary file8 (PDF 257 KB) [file 330_2023_9651_MOESM8_ESM.pdf]

	Gadolinium administration route and agent	Gadolinium dosage/ volume and delay to MRI (hrs)	Post gadolinium sequence/ post processing (MRI system strength)	Number of. independent observers (agreement statistics or range for differing MRI descriptors)	Cochlear grading system/ perilymphatic enhancement (PLE) evaluated	Vestibular grading system and other	Notes on extracting data for meta-analysis
**Attye† (2017 [a]) [18]**	IV gadoterate	0.1 mmol/kg 4.5-5.5 hrs	3D FLAIR VFA (3T)	2 (kappa 0.91)	NA	SURI	
**Attye [α] (2017 [b]) [19]**	IV gadoterate	0.1 mmol/kg 4 hrs	3D FLAIR VFA(3T)	2 (kappa 0.63-1)	Nakashima	Nakashima/ SURI	
**Attye^ (2018) [20]**	IV gadoterate	0.1 mmol/kg 4.5-5.5 hrs	3D FLAIR VFA (3T)	2 (kappa 0.91)	NA	SURI	
**Attye† (2020) [21]**	IV gadoterate	0.1 mmol/kg 4 hrs	3D FLAIR VFA (3T)	2	NA	SURI[β]	
**Barath (2014) [22]**	IV gadobutrol	0.2 mmol/kg 4 hrs	3D FLAIR CFA (3T)	2	Barath	Barath	
**Beckers[α] (2016) [23]**	IV NS	NS 4 hrs	3D FLAIR (3T)	NS	NS	NS	
**Bernaerts (2019) [24]**	IV gadobutrol	0.3 mmol/kg 4 hrs	3D FLAIR CFA (3T)	3 (kappa for all MD 0.85-0.92)	Bernaerts/ PLE	Bernaerts	
**Bernaerts (2022) [25]**	IV gadobutrol	0.1 mmol/kg 4 hrs	3D FLAIR CFA (3T)	2 (kappa 0.76-0.87)	Bernaerts/ PLE[Ω]	Bernaerts	
**Boegle (2021) [26]**	IV gadobutrol	0.1 mmol/kg 4 hrs	3D FLAIR CFA (3T)	3 (Kendalls co-eff W 0.70-0.74)	Authors own grading	Authors own grading	Only leading ear data included when bilateral MD
**Carfrae (2008) [27]**	IV gadodiamide	0.2 mmol/kg 4 hrs	3D GRE-T1 (3T)	1	Authors own grading	NA	

**Chen [λ] (2021) [28]**	IV gadoteridol	0.2 mmol /kg 3.5-4 hrs	3D REAL IR CFA (3T)	2	Nakashima	Nakashima	
**Choi [Π] (2017) [29]**	IV gadobutrol	NS 4 hrs	hT2W-3D FLAIR CFA / HYDROPS (3T)	2 (Kendall's tau 0.767 affected, 0.946 unaffected)	NA	Nakashima	
**Connor[Πα] (2020) [30]**	IV gadoterate	0.3 mmol/kg 4 hrs	3D FLAIR CFA (3T)	2 (ICC 0.97 for quantitative)	NA	Nakashima	
**Conte (2018) [31]**	IV gadoteridol	0.2 mmol/kg 4 hrs	3D FLAIR CFA (3T)	2 (kappa 0.867)	Nakashima /PLE[Ω]	Nakashima[β]	
**Dominguez (2021) [32]**	IV gadobutrol	0.1 mmol/kg 4-5 hrs	3D REAL IR CFA (3T)	2 (kappa 0.59-0.8)	Bernaerts[β]	Nakashima	Applied EH grading in "affected" ear vMD for meta-analysis
**Eliezer (2017) [33]**	IV gadoterate	0.3 mmol/kg 4 hrs	3D FLAIR VFA (3T)	2	Nakashima	Nakashima/ SURI	Results for 2300ms TI used for meta-analysis
**Eliezer (2018) [34]**	IV gadoterate/ IV gadobutrol	0.2 mmol/kg/ 0.1mmol/kg 4 hrs	3D FLAIR VFA (3T)	2	Nakashima	SURI	2 separate groups of n=10 with different gadolinium agents
**Eliezer ^ (2021) [35]**	IV gadobutrol	0.1 mmol/kg 4 hrs	3D FLAIR VFA (3T)	2 (ICC 0.87)	NA	SURI	
**Eliezer[ø] (2022) [36]**	IV gadobutrol	0.1 mmol/kg 4 hrs	3D FLAIR CFA (3T)	2	Khan	Khan	
**Fang[○] ^ (2012) [37]**	IT gadopentate	NS v/v 1:7 saline 24 hrs	3D FLAIR (3T)	2	Authors own grading	Authors own grading	

**Fiorino (2011) [38]**	IT gadobutrol	0.6 ml of 1 mmol/ml v/v 1:7 saline 24 hrs	3D FLAIR CFA (3T)	NS	Authors own grading	Authors own grading	
**Grieve^ (2012) [39]**	IT gadoteridol	0.5 ml of 1 mmol/ml v/v 1:7 saline 24 hrs+_8hr	3D REAL IR (3T)	2	Authors own grading	Authors own grading	
**Grosser (2021) [40]**	IV gadobutrol	0.2 mmol/kg 4 hrs	3D FLAIR CFA 3D REAL IR CFA/ HYDROPS (3T)	NS	Authors own grading	NS	
**Hagiwara (2014) [41]**	IV gadopentate	0.3 mmol/kg 4 hrs	3D FLAIR VFA (3T)	2	Authors own grading	NA	No consensus data so value applied from single observer
**Horii^ (2011) [42]**	IT gadodiamide	1 mmol/ml v/v 1:7 saline 24 hrs	2D FLAIR (3T)	2	Nakashima	Nakashima	
**Imai (2017) [43]**	IV gadoteridol	0.4 mmol/kg 4 hrs	2D FLAIR (3T)	4 (kappa 0.8)	Authors own grading	NA	No consensus data so mode values applied
**Ito^ (2016) [44]**	IV gadopentate	0.1 mmol/kg 4 hrs	3D FLAIR CFA/ HYDROPS (3T)	2	Nakashima	Nakashima (modified)	
**Jasinska (2022) [45]**	IV gadobutrol	0.2 mmol/kg 4 hrs	3D FLAIR CFA (3T)	1	Bernaerts	Barath	
**Kahn (2020) [46]**	IV gadobutrol	0.1 mmol/kg 4 hrs	3D FLAIR CFA (3T)	2 (kappa	Kahn/PLE	Kahn	

				0.84-0.9)			
**Katayama (2010) [47]**	IT gadodiamide	1 ml of 1 mmol/ml v/v 1:7 saline 24 hrs	3D FLAIR CFA and 3D REAL IR CFA (3T)	1	Nakashima	Nakashima	
**Kawai ^ (2010) [48]**	IT gadopentate	v/v 1:7 saline 24 hrs	3D REAL IR CFA (3T)	2	Nakashima$^{\beta}$	Nakashima	
**Kenis (2021) [49]**	IV gadobuterol	0.3 mmol/kg 4 hrs	3D FLAIR CFA (1.5T)	1	Bernaerts/ PLE	Bernaerts	
**Kierig ^ (2019) [50]**	IV gadobutrol	NS 4 hrs	NS (3T)	NS	Barath	Barath (modified)	
**Li (2020) [51]**	IT gadopentate	1ml/ v/v 1:7 saline 24 hrs	3D FLAIR and REAL IR (3T)	1	Nakashima	Nakashima	
**Li (2022) [52]**	IV NS	0.4 ml/kg 6 hrs	3D FLAIR (3T)	2	Authors own grading	NA	
**Lin^ (2021) [53]**	IV gadodiamide	0.1 mmol/kg 4 hrs	3D FLAIR CFA/ HYDROPS-Mi2 (3T)	1	Nakashima	Bernaerts	
**Liu ^ (2015) [54]**	IT gadodiamide	0.4-0.6 ml of 1 mmol/ml v/v 1:7 saline 24 hrs	3D FLAIR (3T)	1	Nakashima	Nakashima	
**Mainnemarre ^ (2020) [55]**	IV gadobutrol	0.1 mmol/kg 4 hrs	3D FLAIR CFA (3T)	2 (ICC 0.93)	Khan	Khan	
**Morimoto (2017) [56]**	IV gadodiamide IT gadopentatate	IV: 0.1 mmol/kg 4 hrs IT: 1 mmol/ml	3D FLAIR CFA and 3D REAL IR CFA/	1	Nakashima	Nakashima	One patient removed from MA due to overlapping data with Okazaki et al

		v/v 1:7 saline 24 hrs	HYDROPS2 (3T)				
**Morimoto* (2020) [57]**	IV gadodiamide	0.1 mmol/kg 4 hrs	3D FLAIR CFA and 3D REAL IR CFA HYDROPS2 (3T)	2	Nakashima	Nakashima[β]	
**Morita[ø] (2020) [58]**	IV gadobutrol	0.1 mmol/kg 4 hrs	3D FLAIR CFA/ HYDROPS (3T)	2	Nakashima	Nakashima	
**Murofushi (2020) [59]**	IV gadobutrol	0.1 mmol/kg 4 hrs	3D FLAIR/ HYDROPS (3T)	NS	Nakashima	Nakashima	
**Naganawa* (2012[a]) [60]**	IV gadodiamide	0.1 mmol/kg 4 hrs	3D FLAIR CFA/ HYDROPS (3T)	2	Nakashima	Nakashima	
**Naganawa*^[a] (2012 [b]) [61]**	IV gadodiamide	0.1 mmol/kg 3.5-4.5 hrs	3D FLAIR CFA/ HYDROPS (3T)	2 (kappa 0.924)	Nakashima	Nakashima	
**Naganawa*[a] (2013) [62]**	IV gadodiamide	0.1 mmol/kg 4 hrs	hT2W-3D FLAIR CFA/ HYDROPS Mi2 (3T)	1	Nakashima	Nakashima	
**Nahmani ^ (2020) [63]**	IV gadobutrol	0.1 mmol/kg 4 hrs	3D FLAIR CFA/VFA(3 T)	2 (kappa 0.26/1.0 for differing MRI sequences)	PLE	SURI and fusion of utricle/ saccule	Consensus data only available for constant flip

							angle so used for meta-analysis
**Nakada[ø] (2014) [12]**	IV gadodiamide	0.1 mmol/kg 4 hrs	3D FLAIR/ HYDROPS (3T)	1	Nakashima	Nakashima	
**Noh[π] (2021) [64]**	IV gadopentetate	NS 4 hrs	3D FLAIR CFA/ HYDROPS-Mi2 (3T)	NS	Nakashima	Nakashima	
**Oh[ø] (2021) [65]**	IV gadoterate	0.1 mmol/kg 4 hrs	3D FLAIR CFA/ HYDROPS-Mi2 (3T)	1	Nakashima	Nakashima	
**Okazaki[λ] (2017) [66]**	IV gadodiamide	0.1 mmol/kg 4 hrs	3D FLAIR and 3D REAL IR/ HYDROPS2 (3T)	1	Nakashima	Nakashima	
**Pai (2020) [67]**	IV gadoterate	0.2 mmol/kg 4 hrs	3D FLAIR CFA (3T)	1	Nakashima	Nakashima/ SURI/fusion of utricle/ saccule	
**Pakdaman (2016) [68]**	IV gadopentetate	0.1 mmol/kg 4 hrs	3D FLAIR CFA/ 3D MIP (3T)	1	NA	Grade 2 Nakashima	
**Perez-Fernandez[α] (2019) [69]**	IV gadobutrol	0.1 mmol/kg 4 hrs	2D FLAIR and 3D FLAIR CFA and	1	Authors own grading	Bernaerts variant	

			REAL IR<br>VFA (3T)				
**Pyykko*^ (2013) [70]**	IV gadodiamide gadopentetate	0.1 mmol/kg<br>4 hrs	hT2W-3D FLAIR CFA (3T)	NS	Nakashima	Nakashima	
**Qin^ (2021) [71]**	IT NS	0.5 ml of 1 mmol/ml v/v 1:7 saline<br>24 hrs	3D FLAIR (3T)	2	Authors own grading	Authors own grading	
**Quatre (2019) [72]**	IV gadoterate	0.1 mmol/kg<br>4.5-5.5 hrs	3D FLAIR (3T)	2<br>(kappa 0.91)	NA	SURI	
**Sano* (2012) [73]**	IV gadodiamide	0.1 mmol/kg<br>4 hrs	hT2W-3D FLAIR CFA (3T)	1	Nakashima	Nakashima	
**Shi (2018) [74]**	IV gadoteridol	0.2 mmol/kg<br>4 hrs	3D REAL IR (3T)	2	Nakashima/ PLE	Nakashima	
**Shimono^ (2013) [75]**	IV gadodiamide	0.3 mmol/kg<br>4 hrs	hT2W-3D FLAIR/ HYDROPS (3T)	1	Nakashima	Nakashima	
**Shiraishi (2020) [76]**	IV gadoterate	0.1 mmol/kg<br>4 hrs	hT2W-3D FLAIR/ HYDROPS (3T)	2	Khan	Khan	
**Sousa[Π] (2021) [77]**	IV NS	NS<br>4 hrs	hT2W-3D FLAIR/ HYDROPS (3T)	1	NS	Nakashima	
**Suarez Vega[α] (2019) [78]**	IV NS	NS<br>4 hrs	3D FLAIR<br>3D REAL IR (3T)	1	NS	NS	

**Sun[a] (2017) [13]**	IT NS	v/v 1:7 saline<br>24 hrs	3D REAL IR (3T)	1	Nakashima	NS	
**Tagaya* (2011) [79]**	IV gadoteridol	0.2 mmol/kg<br>4 hrs	3D FLAIR CFA<br>3D REAL IR (3T)	1	Nakashima	Nakashima	
**Tanigawa^ (2011) [80]**	IV gadodiamide	0.1 mmol/kg<br>4 hrs	3D FLAIR (3T)	NS	Authors own grading	Authors own grading	
**Tunon Gomez^ (2017) [81]**	IT gadoterate	0.45-0.9 ml of 0.5 mmol/ml<br>v/v 1:7 saline<br>24-28 hrs	3D REAL IR CFA (3T)	2	Authors own grading	Authors own grading	
**Van Steekelenburg[○] (2016) [82]**	IV gadoterate	15mmol<br>4 hrs	3D FLAIR CFA (3T)	2<br>(kappa 0.9-0.93)	Barath/PLE	Bernaerts	
**Vanspauwen[α] (2016) [83]**	IV NS	"double dose"<br>4 hrs	3D FLAIR (3T)	NS	NS	NS	
**Wu (2016) [84]**	IT NS	NS<br>24 hrs	3D FLAIR<br>3D REAL IR (3T)	1	Nakashima	Nakashima	
**Xie (2021) [85]**	IT gadopentetate	0.5ml of 1 mmol/ml<br>v/v 1: 7 saline<br>24 hrs	3D FLAIR VFA (3T)	2	Nakashima	Nakashima	
**Yoshida* (2018) [86]**	IV gadodiamide	0.1 mmol/kg<br>4 hrs	hT2W-3D FLAIR/ HYDROPS (3T)	1	Nakashima	Nakashima	
**Yoshida (2021) [87]**	IV gadobutrol	0.1 mmol/kg<br>4 hrs	hT2W-3D FLAIR CFA/	>=2	Nakashima	Nakashima	

			HYDROPS (3T)				

† Overlapping normal control ears with other studies so these controls removed from meta-analysis

β Additional MRI descriptors not included in table or meta-analysis since applied in ≤4 eligible studies

α Overlapping data set with other studies and excluded from meta-analysis

Ω Not possible to create 2x2 tables for PLE so excluded from meta-analysis

^ Clinical classification of MD not stated, defined as a combination of different criteria, or applied an alternative clinical classification so excluded from clinical criteria subgroup meta-analysis

λ Additional data obtained from authors

π Vestibular grading derived from quantitative measures

ø Applied maximum EH grading in either ear when no localising audio vestibular symptoms (eg vestibular MD or vestibular migraine)

○ Control ears are combined asymptomatic MD ears and other/normal ears so excluded from control group metaregression

*Data from same institution as other studies during similar time period but no evidence of overlapping data

Abbreviations

NS: Not stated; **3D FLAIR**: 3D Fluid attenuation inversion recovery; **hT2W-3D FLAIR**: Heavily T2w 3D-FLAIR; **3D REAL IR**: 3D REAL inversion recovery; **VFA**: Variable flip angle; **CFA**: Constant flip angle

HYDROPS: Hybrid of reversed image of positive endolymph signal and native image of positive perilymph signal. Subtraction of a positive endolymph image (PEI) from a heavily T_2-weighted 3D-fluid attenuated inversion recovery or positive perilymph image (PPI)

HYDROPS-2: Hybrid of reversed image of MR cisternography and positive perilymph signal by heavily T_2-weighted 3D-FLAIR

HYDROPS-Mi2: HYDROPS image multiplied by T_2-weighted magnetic resonance cisternography

MIP: maximum intensity projection

PLE: Increased ipsilateral cochlear perilymphatic enhancement

Supplementary 6: MRI gadolinium enhancement technique, sequence, post processing, number of observers and grading scale
