# Supplementary material for: Delayed post gadolinium MRI descriptors for Meniere’s disease: a systematic review and meta-analysis
Source: Eur Radiol. 2023 May 12;33(10):7113–35. doi: 10.1007/s00330-023-09651-8 (PMC10511628; doi:10.1007/s00330-023-09651-8)
Supplement: Supplementary file 9 — Supplementary file9 (PDF 118 KB) [file 330_2023_9651_MOESM9_ESM.pdf]

Grading scale		Nakashima et al (95)	Barath et al (22)	SURI (19)	Bernaerts et al (24)	Kahn et al (46)
**MRI descriptor**	**Any vestibular EH**	Vestibular 1/2	Vestibular 1/2	Yes	Vestibular 1/2/3	Saccular 1/2 or utricular 1/2
	**>33% area of ES relative to total vestibular fluid area (figure 2f)**	Vestibular 1/2	NA	NA	NA	NA
	**>50% area of ES relative to total vestibular fluid area (figure 2g)**	Vestibular 1/2	Vestibular 1/2	NA	NA	NA
	**SURI or higher vestibular grade (figure 2e)**	NA	NA	Yes	Vestibular 1/2/3	Saccular 1/2
	**Fused utricle and saccule (figure 2f)**	NA	NA	NA	Vestibular 2/3	NA
	**Enhancing PS of the vestibule not visible (figure 2g)**	NA	Vestibular 2	NA	Vestibular 3	Utricular 2
	**Any cochlear EH**	Cochlear 1/2	Cochlear 1/2	NA	Cochlear 1/2	Cochlear
	**Highest grade cochlear EH (figure 2g)**	Cochlear 2	Cochlear 2	NA	Cochlear 2	NA
	**Increased ipsilateral PLE (figure 2h)**	NA	NA	NA	NA	NA

Individual MRI descriptors were selected for analysis if they could be applied to at least 4 eligible studies. Hence infrequently applied MRI descriptors (ruptured/absent saccule, vestibular aqueduct visibility, ampullary herniation to the lateral semi-circular canal and contact of the endolymphatic space with the oval window) (19) (21) (31) (32) were not analysed.

Supplementary 7: Derivation of MRI descriptors from grading scales
