# Supplementary material for: Delayed post gadolinium MRI descriptors for Meniere’s disease: a systematic review and meta-analysis
Source: Eur Radiol. 2023 May 12;33(10):7113–35. doi: 10.1007/s00330-023-09651-8 (PMC10511628; doi:10.1007/s00330-023-09651-8)
Supplement: Supplementary file 10 — Supplementary file10 (PDF 109 KB) [file 330_2023_9651_MOESM10_ESM.pdf]

		No. studies	Chi squared test for equality of sensitivities (p value)	Chi squared test for equality of specificities (p value)	Correlation of sensitivity and false positive rate (rho)
**MRI Descriptors**	**Any vestibular EH**	53	<2E-16	<2E-16	0.3 (0.03, 0.53)
	**>33% area of ES relative to total vestibular fluid area**	28	2.46E-13	<2E-16	0.07 (-0.32,0.43)
	**>50% area of ES relative to total vestibular fluid area**	23	6.73E-05	2.15E-09	-0.07 (-0.47,0.36)
	**SURI or higher vestibular EH grade**	20	<2E-16	3.79E-14	0.37 (-0.11,0.7)
	**Fused utricle and saccule**	8	0.00019	0.24	0.31 (-0.5, 0.83)
	**Enhancing PS of the vestibule not visible**	10	4.19E-05	0.59	0.07 (0.22,0.38)
	**Any cochlear EH**	43	<2E-16	<2E-16	0.46 (0.19, 0.67)
	**Highest grade cochlear EH**	26	<2E-16	1.2E-11	0.64 (0.34, 0.83)
	**Increased ipsilateral PLE**	6	2.26E-07	0.45	-0.54 (-0.94,0.48)
	**Increased ipsilateral PLE or any EH**	3	NA	NA	NA
	**Any EH**	55	<2E-16	<2E-16	0.48 (0.25, 0.67)

NA Not applicable due to small number of eligible studies

Supplementary 8: Heterogeneity of sensitivity and specificity for the MRI descriptors
